# Emerging role of MYB transcription factors in cancer drug resistance

**DOI:** 10.20517/cdr.2023.158

**Published:** 2024-04-30

**Authors:** Bernhard Biersack, Michael Höpfner

**Affiliations:** ^1^Organic Chemistry Laboratory, University of Bayreuth, Bayreuth 95440, Germany.; ^2^Institute for Physiology, Charité-Universitätsmedizin Berlin, Corporate Member of Freie Universität Berlin and Humboldt Universität zu Berlin, Berlin 10117, Germany.

**Keywords:** Anticancer drugs, MYB, transcription factors, oncogenes, drug resistance, cancer resistance mechanisms, MYB-targeting drugs

## Abstract

Decades ago, the viral myeloblastosis oncogene *v*-*myb* was identified as a gene responsible for the development of avian leukemia. However, the relevance of MYB proteins for human cancer diseases, in particular for solid tumors, remained basically unrecognized for a very long time. The human family of MYB transcription factors comprises MYB (c-MYB), MYBL2 (b-MYB), and MYBL1 (a-MYB), which are overexpressed in several cancers and are associated with cancer progression and resistance to anticancer drugs. In addition to overexpression, the presence of activated MYB-fusion proteins as tumor drivers was described in certain cancers. The identification of anticancer drug resistance mediated by MYB proteins and their underlying mechanisms are of great importance in understanding failures of current therapies and establishing new and more efficient therapy regimens. In addition, new drug candidates targeting MYB transcription factor activity and signaling have emerged as a promising class of potential anticancer therapeutics that could tackle MYB-dependent drug-resistant cancers in a more selective way. This review describes the correlation of MYB transcription factors with the formation and persistence of cancer resistance to various approved and investigational anticancer drugs.

## INTRODUCTION

Cancer drug resistance has become a prolific and important field of cancer research. However, the underlying mechanisms are complex and still only incompletely understood. ATP-binding cassette (ABC) transporters are prominent examples of ATP-dependent drug efflux pumps mediating multi-drug resistance with clinical potential; nevertheless, ABC-transporter inhibition has achieved no relevance for daily clinical practice so far. Less energy-consuming resistance mechanisms often arise and circumvent ABC-transporter blocking^[1]^. Transcription factors are also well-established resistance mediators in cancer diseases, but except for hormone receptors, they are difficult to target by candidate drugs^[2]^. Generally, transcription factors are regulated by, and interact with, several other (co-)factors, forming well-balanced transcriptional complexes with activating or repressing properties, which need to be thoroughly elucidated to understand any oncogenic effects^[3]^. The expression of transcription factors itself is highly regulated, and their transcripts can be suppressed by non-coding RNAs via direct interaction^[4]^. Post-translational modifications of transcription factors such as phosphorylation by protein kinases and acetylation by histone acetyltransferases, as well as their removal by phosphatases and histone deacetylases, contribute significantly to the activity, stability, and nuclear translocation of transcription factors, usually in cooperation with other proteins such as heat-shock proteins (Hsps) and ubiquitin ligases^[2,5]^.

Oncogenic transcription factors can be subdivided into several classes based on their constitution and activities^[6]^. MYC is the gene product of the cellular myelocytomatosis oncogene and one of the most investigated transcription factors involved in the formation and progression of blood cancers and solid tumors^[7-9]^. The MYB (myeloblastosis) family of transcription factors has meanwhile also garnered increased importance as a driver of cancer progression and resilience^[10,11]^. In fact, MYC expression is tightly regulated by MYB proteins, which highlights one important aspect of the manifold roles of MYB proteins in cancer-associated processes^[12]^. Initially, the viral myeloblastosis oncogene *v*-*myb* was identified as a gene of the avian myeloblastosis retrovirus (AMV) responsible for the development of myeloid leukemia in birds^[13]^. The v-MYB protein is truncated when compared with the vertebrate MYB proteins and lacks certain regulatory domains^[14]^. The human MYB protein family comprises the original cellular MYB (c-MYB), as well as the MYB proto-oncogene like proteins MYBL1 (a-MYB) and MYBL2 (b-MYB)^[15]^. These MYB proteins have a highly conserved N-terminal helix-turn-helix DNA-binding domain (DBD), a central trans-activating domain (TAD), and a C-terminal negative regulatory domain (NRD)^[16,17]^. All MYB forms bind to a specific DNA sequence [PyAAC(G/T)G] as canonical MYB binding site to exert their transcription regulatory properties^[18]^. MYB binds more than ten thousand promoters in cancer cells and regulates genes involved in cell cycle [cyclins and cyclin-dependent kinases (CDKs)] and cell death [survivin (BIRC5), B-cell lymphoma 2 (BCL2), ataxia telangiectasia and Rad3 related protein (ATR)], as well as protein kinases [insulin-like growth factor 1 receptor (IGF-1R), KIT, polo-like kinase 1 (PLK1)] and growth factors [vascular endothelial growth factor (VEGF)]^[10,19]^. The trans-activating properties of MYB are modulated by interaction with distinct cofactors such as cAMP response element-binding protein (CREB)-binding protein (CBP), the histone acetyltransferase p300, CCAAT/enhancer-binding protein β (C/EBPβ), as well as through post-translational modifications such as ubiquitination, sumoylation, acetylation, and phosphorylation^[20,21]^.

All three human MYBs are proven promotors of human cancers, albeit via differing mechanisms and in selected cancers. MYB overexpression was observed in leukemia, gastrointestinal cancers (colorectal cancer and pancreatic cancer), and breast cancer. In adenoid cystic carcinoma (ACC), tumor-promoting fusion proteins of MYB and MYBL1 with nuclear factor IB (NFIB) were identified^[10]^. MYBL2 regulates cell cycle, apoptosis, and epithelial-to-mesenchymal transition (EMT), and is overexpressed in leukemia and several solid tumors (e.g., breast, lung, colorectal and gallbladder cancer, hepatocellular and esophageal squamous cell carcinoma, glioma, and neuroblastoma)^[22]^. MYB proteins coincidentally exhibit tumor-suppressing properties, too. Moreover, their effects are not confined to cell cycle and apoptosis/cell death-associated mechanisms. The tumor response to hypoxia conditions is mediated by MYB and MYBL2 and their interaction with the ubiquitin ligase protein of the von Hippel-Lindau (*pVHL*) gene and hypoxia-inducible factors (HIFs)^[23,24]^. The interaction of MYB with pVHL is regulated by the crucial corepressor MYB-binding protein 1A (MYBBP1A)^[25]^. In addition, MYB, MYBBP1A, and MYBL2 control tumor metabolism and glycolysis^[26-28]^. MYBL2 also plays a role in chromosome stability and forms a complex with clathrin and filamin in functional mitotic spindles^[29]^. Cancer stemness, angiogenesis, and autocrine/paracrine signaling by upregulation of growth factors are also promoted by MYB proteins^[30]^.

This review summarizes and discusses the current knowledge of the influence of MYB proteins on the resistance to anticancer drugs that are clinically approved or in clinical trials. A concluding section about promising inhibitors of MYB activity with the potential to overcome drug resistance rounds out this review.

## MYB PROTEINS AND CANCER DRUG RESISTANCE

### Resistance to chemotherapy

The introduction of the first chemotherapeutics into clinical practice already happened in the 1950s, and their ability to cure childhood leukemia and Hodgkin lymphoma since the 1960s was a milestone^[31]^. Today, the vast majority of cancer patients still receive chemotherapy to control the disease despite the progress in various other fields of oncology such as surgery, radiotherapy, stem cell therapy, targeted therapy, and immune therapy^[32]^. Resistance to chemotherapeutics poses a severe problem and new therapies are sought to overcome cancer resistance^[33]^. Notably, MYB transcription factors contribute significantly to chemotherapy resistance either as overexpressed oncogenes or as downregulated tumor suppressors, which mirrors the Janus-like character of MYB proteins in cancer progression and resistance formation.

#### Resistance to platinum complexes

Platinum complexes (cisplatin, carboplatin, and oxaliplatin) belong to the most salient chemotherapeutics for the treatment of solid tumors^[34]^. The therapy of testicular germ cell cancer with cisplatin shows curing rates of 95%. However, resistant forms of this and other cancers such as ovarian cancers treated with platinum complexes pose a considerable clinical problem^[35,36]^. Cisplatin resistance is mediated by MYB proteins. Increased expression of MYB was detected in cisplatin-resistant colorectal carcinoma (CRC) cells, and treatment with a *c-myb* antisense oligonucleotide sensitized these resistant cells to cisplatin, indicating a significant role of MYB in cisplatin resistance of CRCs^[37]^. In ovarian cancer (OC) patients, high levels of MYB were associated with bad prognosis and poor progression-free survival, especially in African American people^[38]^. MYB overexpression led to cisplatin resistance in OC cells by activation of Nuclear factor k-light-chain-enhancer of activated B cells (NF-κB) and signal transduction activator of transcription 3 (STAT3) signaling, while MYB silencing and MYB suppression sensitized OC cells to cisplatin^[39]^. Increased STAT3 activity by upregulated MYB was also observed in cholangiocarcinoma, pancreatic cancer, and salivary ACC^[40-42]^. However, the documented anti-metastatic properties of MYB in breast cancers were associated with anti-inflammatory processes such as NF-κB suppression, indicating the existence of different forms of MYB-mediated NF-κB function. Although increased MYB levels promote breast cancer cell proliferation, the role of MYB in breast cancer is more complex and apparently context-dependent, with differences between proliferation and metastasis stages^[43]^. In terms of non-coding RNAs, upregulation of the oncomir miR-21 by increased MYB was involved in cisplatin resistance of OC cells, which was accompanied by activation of Wnt signaling and EMT, and suppression of β-catenin and the tumor suppressor miR-200c. Accordingly, MYB-overexpressing and cisplatin-resistant OC xenografts were successfully re-sensitized to cisplatin by anti-miR-21^[44]^. It is noteworthy that the combination of cisplatin with the CEBPβ-regulated tumor suppressing long non-coding RNA (lncRNA) LOC102724169 showed synergistic effects in chronically stressed OC xenografts by suppression of MYB and phosphoinositide 3 kinase (PI3K)/protein kinase B (AKT) signaling^[45]^. Upregulation of PI3K-AKT signaling by MYB was also observed in non-malignant cochlear hair cells, which prevented cisplatin-mediated apoptosis and cell demise associated with reduced chemotherapy-based ototoxicity side effects *in vivo*^[46]^. This example vividly shows the natural function of MYB in the protection of cells from harmful xenobiotics. The human UM-HACC-2A cell line was established as a MYB-NFIB-positive ACC cell line, which also showed increased MYB, epidermal growth factor receptor (EGFR/ErbB1), and E-cadherin levels. These ACC cells were resistant to cisplatin, but sensitive to paclitaxel, providing evidence of a MYB-NFIB-mediated resistance mechanism to DNA-targeting drugs^[47]^. In gastric cancer, MYBL2 activated ubiquitin-conjugating enzyme E2 C (UBE2C), leading to cisplatin resistance and cancer progression based on downregulated apoptosis and DNA damage response^[48]^. Other MYB-like proteins have revealed tumor-suppressing properties in the context with cisplatin therapy. The protein MYSM1 (MYB-like, SWIRM, and MPN domains 1) is a histone H2A deubiquitinase that augments the activity of cisplatin in triple-negative breast cancer (TNBC) cells by suppression of p90 ribosomal S6 kinase 3 (RSK3) and induction of apoptosis mediated by activated BCL2 antagonist of cell death (BAD)^[49]^. The tumor suppressor cyclin D binding MYB-like transcription factor 1 (DMTF1) stabilizes p53, and downregulation of DMTF1 was associated with cisplatin resistance in breast cancer cells^[50]^.

Carboplatin is a surrogate of cisplatin because of its similar activity profile but low toxicity. Spheroids of epithelial ovarian cancer (EOC) cells depended on the dimerization partner, RB-like, E2F and multi-vulval class B (DREAM) repressor complex induced by dual specificity tyrosine-phosphorylation-regulated kinase 1A (Dyrk1A). Inhibition of Dyrk1A by harmine or INDY blocked DREAM and promoted MYBL2-MuvB (MMB) complex formation, which sensitized EOC spheroid cells to carboplatin treatment^[51]^. Thus, the combination of carboplatin with Dyrk1A inhibitors appears to be promising for EOC therapy and other regulators of DREAM might turn out to be relevant anticancer drug targets in the future.

Because CRCs are usually cisplatin-resistant, the more potent complex oxaliplatin is applied for CRC treatment in combination with 5-fluorouracil (5-FU) and folic acid (FOLFOX therapy). The interplay of MYB and CREB with p300 is important for gastrointestinal homeostasis and CRC formation. Transcription via p300-CREB enhanced oxaliplatin resistance in CRC by upregulation of the ABC transporter multidrug resistance-associated protein 2 (MRP2), while p300-MYB was more involved in gastrointestinal differentiation^[52]^. The role of MYBL2 in oxaliplatin resistance of CRC cells was studied and MYBL2 was shown to induce the expression of the lncRNA CCAT1, which led to upregulation of suppressor of cytokine signaling 3 (SOCS3) and resistance to oxaliplatin *in vitro* and *in vivo*^[53]^. Resistance of CRCs to cisplatin and oxaliplatin was also associated with p53 absence in p53-knockout CRC cells. MYB expression was strongly upregulated in the p53-knockout cells compared with p53-wildtype cells, which might explain the multidrug-resistant phenotype of the p53-negative CRC cells and the poor prognosis for CRC with overexpressed MYB^[54]^. MYB also prevented apoptosis induction by cisplatin and oxaliplatin in CRC cells via increased expression of NADPH oxidase 1 (NOX1) followed by induction of pro-survival p38-mitogen-activated protein kinase (MAPK) signaling^[55]^. These results suggest that targeting the NOX1-p38 axis via specific inhibitors can become a suitable strategy for overcoming platinum resistance in CRC. All in all, the effects of MYB transcription factors on platinum drug efficacy and resistance in various solid tumors are well documented and provide suitable starting points for the design of optimized therapies. Table 1 summarizes the platinum resistance mechanisms based on MYB proteins.

**Table 1:** Platinum resistance of cancers mediated by MYB proteins

**Drug**	**Cancer/cell line**	**Resistance mechanism**
Cisplatin	SWI480DDP and SW620DDP CRC	Increased MYB
Cisplatin	ES2 and OVCAR3 OC	NF-κB and STAT3 activation by MYB overexpression
Cisplatin	ES2 and OVCAR3 OC	Increased miR-21 by MYB overexpression, activation of Wnt and EMT, suppression of β-catenin and miR-200c
Cisplatin	SKOV3 OC	Upregulated MYB and PI3K/AKT by low lncRNA LOC102724169 (under chronic stress)
Cisplatin	MYB-NFIB-positive UM-HACC-2A ACC	Increased MYB, EGFR and E-cadherin
Cisplatin	MKN45, HGC-27, SNU-1 and AGS gastric cancer	MYBL2-activated UBE2C, downregulated apoptosis and DNA damage response
Cisplatin	A2780 and SKOV3 OC	High MYBL2 and CDCA8
Cisplatin	MDA-MB-231 and Hs578T TNBC	MYSM1 suppression, upregulated RSK3, BAD (apoptosis) suppression
Cisplatin	MCF-7 breast cancer	DMTF1 downregulation, p53 inactivation
Carboplatin	OVCAR8, HEY and patient-derived OC	Dyrk1A induced DREAM and suppressed MMB
Oxaliplatin	SW480 and resistant SW480R CRC	Upregulated SOCS3 by MYBL2-induced lncRNA CCAT1 expression
Cisplatin, oxaliplatin	p53-KO HCT-116 CRC	Increased MYB
Cisplatin, oxaliplatin	CRC with exogenous MYB	MYB-mediated apoptosis suppression via increased NOX1 and p38
Cisplatin	Lung adenocarcinoma patient samples	Low MYBL2

CRC: Colorectal carcinoma; OC: ovarian carcinoma; NF-κB: nuclear factor k-light-chain-enhancer of activated B cells; STAT3: signal transduction activator of transcription 3; EMT: epithelial-to-mesenchymal transition; PI3K: phosphoinositide 3 kinase; AKT: protein kinase B; NFIB: nuclear factor IB; ACC: adenoid cystic carcinoma; EGFR: epidermal growth factor receptor; UBE2C: ubiquitin-conjugating enzyme E2 C; CDCA8: cell division cycle-associated 8; TNBC: triple-negative breast cancer; BAD: BCL2 antagonist of cell death; DMTF1: D binding MYB-like transcription factor 1; DREAM: dimerization partner, RB-like, E2F and multi-vulval class B; MMB: MYBL2-MuvB; SOCS3: suppressor of cytokine signaling 3; NOX1: NADPH oxidase 1.

#### Resistance to other DNA-targeting drugs

The anthracyclines daunorubicin and doxorubicin are natural products of *Streptomyces* strains discovered in the 1960s. They soon became valuable anticancer drugs as topoisomerase II inhibitors and producers of reactive oxygen species (ROS), but several resistance mechanisms have limited their application^[56]^. An early study from 1991 stated the correlation between MYB expression and doxorubicin resistance in LoVo/Dx CRC cells, which showed distinctly higher MYB mRNA levels than doxorubicin-sensitive LoVo cells^[57]^. In addition, the suppression of MYB in acute lymphoblastic leukemia (ALL) cells transfected with doxycycline-regulated lentiviral sh-c-Myb sensitized these cells to treatment with DNA-targeting drugs (doxorubicin and the antimetabolite 6-mercaptopurine) by downregulation of anti-apoptotic BCL2^[58]^. MYB promoted leukemia stem cell phenotype via interaction with the insulin-like growth factor 2 mRNA-binding protein 1 (IGF2BP1), and IGF2BP1 knockdown sensitized leukemia cells to the DNA-targeting drugs doxorubicin, cyclophosphamide, and cytarabine. The enforced expression of IGF2BP1 in leukemia cells led to doxorubicin resistance^[59]^. Notably, targeting IGF signaling has meanwhile been identified as a promising strategy to treat MYB-NFIB ACC^[10]^. High levels of MYB in osteosarcoma (OS) are correlated with poor prognosis and resistance, and MYB-knockout in OS cells provoked a sensitization to treatment with doxorubicin and the antimetabolite methotrexate^[60]^. MYB also prevented apoptosis induction by doxorubicin in CRC cells via activation of the NOX1-p38 axis^[55]^. The MYB-NOX1-p38 axis is also involved in resistance to platinum drugs, which underlines the role of this mechanistic pathway in the performance of different DNA-damaging drugs in CRC. Breast cancer with high MYBL2 expression is associated with poor prognosis and a basal-like subtype. Increased ectopic MYBL2 expression in *hTERT*-immortalized mammary epithelial cells sensitized these cells to treatment with topoisomerase II inhibitors such as doxorubicin by induction of G2/M phase-associated genes, while analogously modified basal-like breast tumor cells remained less responsive^[61]^. In addition, MYBL2-transfected CTLL-2 cytotoxic T-cells upregulated BCL2 and were resistant to doxorubicin-mediated apoptosis^[62]^. The p53-interacting MYB-like factor cyclin D binding myb-like protein 1 (Dmp1) was identified as a prognostic factor in cancers^[63]^. How far anthracycline resistance is mediated by Dmp1 in cancer cells remains to be elucidated. However, in non-malignant fibroblasts, toxic effects by daunorubicin and doxorubicin were diminished by NF-κB activation followed by suppression of the Dmp1 promoter, and *Dmp1-/-* cells were resistant to anthracyclines^[64]^.

The topoisomerase II inhibitor etoposide is a semi-synthetic derivative of podophyllotoxin, a natural lignan isolated from American mayapple (*Podophyllum peltatum*) and, thus, differs structurally from anthracyclines. After its first synthesis in 1966, etoposide was approved for cancer therapy by the FDA in 1983; however, etoposide resistance was soon observed^[65,66]^. MYB expression in HEK-293 cells led to resistance to etoposide-mediated apoptosis. Slug expression was induced by binding of MYB to the *slug* gene and upregulation of pro-metastatic Slug (also known as Snail2), a key regulator of EMT partially contributed to etoposide resistance^[67]^. Analogously to increased doxorubicin activity, MYBL2 expression in *hTERT*-immortalized mammary epithelial cells augmented the cytotoxic activity of etoposide^[61]^. In fibrosarcoma cells, the upregulation of DNA replication and repair regulating genes, including MYBL2, led to resistance to doxorubicin, etoposide, cytarabine, and mafosfamide^[68]^. Resistance of CRC cells to doxorubicin, etoposide, and 5-FU was associated with p53 deletion, leading to strongly upregulated MYB expression^[54]^. Together with the observed CRC resistance to cisplatin and oxaliplatin, the tumor suppressor p53 (known as the “guardian of the genome”) appears to be essential for CRC sensitivity to DNA-targeting drugs of different compound and mechanistic classes.

The DNA-alkylating cyclic triazene temozolomide is approved for the therapy of glioblastoma. Various DNA repair mechanisms, epigenetic mechanisms, and oncogenic signaling pathways contribute to temozolomide resistance in glioblastoma^[69]^. The zinc finger E-box-binding homeobox 1 (ZEB1)/miR-200c/MYB axis plays a crucial role in temozolomide resistance. High levels of ZEB1 downregulated tumor suppressor miR-200c, and low miR-200c induced MYB expression in primary glioblastoma cells, followed by increased O-6-methylguanine DNA methyltransferase (MGMT) expression and DNA repair^[70]^. This mechanism was also responsible for cisplatin resistance in OC cells and vital for DNA adduct repair^[44]^. Conversely, MYB could also activate miR-200, whereas ZEB1 could directly suppress MYB expression in breast cancer cells, which suggests a context-dependent role of MYC in certain cancers^[71,72]^. Suppression of MYB mRNA by miR-107 revealed a possible mechanism of acute myeloid leukemia (AML) resistance to the antileukemic nucleoside cytarabine in AML cell lines and patient samples, which indicates a tumor suppressor role of MYB in the interplay with miR-107^[73]^.

The phthalazinone-based poly (ADP-ribose) polymerase (PARP) inhibitor olaparib is approved as first-line therapy for advanced OC^[74]^. High MYBL2 levels led to increased cell division cycle-associated 8 (CDCA8) expression and drug resistance. Conversely, MYBL2 knockdown and CDCA8 silencing sensitized A2780 and SKOV3 OC cells to olaparib and cisplatin^[75]^. The formation of castration-resistant prostate cancer (CRPC) was mediated by induction of MYB upon androgen deprivation, leading to increased DNA damage response and reduced olaparib sensitivity via the MYB-topoisomerase 2-binding protein 1 (TopBP1)-ATR-checkpoint kinase 1 (CHK1) axis. MYB silencing and the CHK1 inhibitor AZD7762 showed synergistic effects with olaparib in androgen receptor (AR)-positive and -negative prostate cancer cells^[76]^.

Iron-chelating bleomycins (natural metallo-glycopeptides of *Streptomyces verticillus*) cause oxidative DNA damage upon ROS formation. Blenoxane (60% bleomycin A2, 30% bleomycin B2) is a clinically relevant anticancer drug for the treatment of Hodgkin lymphoma, testicular cancer, and head-and-neck cancer^[77]^. AP endonuclease (Ape1/ref-1) is an activator of MYB binding to DNA. Increased Ape1/ref-1 expression stimulated DNA base excision repair, leading to bleomycin resistance in germ cell tumor cells^[78]^. The tetrapyrrole chlorin e6 is a photosensitizer approved for photodynamic therapy that cleaves DNA by ROS formation^[79]^. MYBL2 knockdown in CRC cells led to resistance to chlorin e6-mediated photodynamic therapy by activation of NF-κB and increased expression of the ABC transporter ABCG2^[80]^. The suppressing effect of MYBL2 on ABCG2 is noteworthy because ABCG2 [alternatively referred to as breast cancer resistance protein (BCRP)] is an important drug efflux transporter that eliminates several topoisomerase inhibitors, leading to multidrug resistance^[81]^. Table 2 summarizes the MYB-mediated resistance mechanisms to non-platinum DNA-targeting drugs.

**Table 2:** Cancer resistance to non-platinum DNA-targeting drugs mediated by MYB proteins

**Drug**	**Cancer/cell line**	**Resistance mechanism**
Doxorubicin	LoVo/Dx CRC	Increased MYB
Doxorubicin, 6-mercaptopurin	697 pre B-ALL	MYB and BCL2 expression
Doxorubicin, cyclophosphamide, cytarabine	MOLT16 leukemia	Stem cell phenotype via expression of MYB and IGF2BP1
Doxorubicin, methotrexate	SAOS-2 LM5 and 143B OS	Upregulated MYB
Doxorubicin	CRC	MYB suppressed apoptosis via enhanced NOX1 and p38-MAPK
Doxorubicin, etoposide	Basal-like breast tumor cells	Increased ectopic MYBL2 expression
Doxorubicin	CTLL-2 cytotoxic T-cells	MYBL2 transfection upregulated BCL2 and apoptosis resistance
Doxorubicin, daunorubicin	Fibroblasts	NF-κB activation, suppression of the *Dmp1* promoter
Etoposide	HEK-293	MYB expression upregulated Slug
Doxorubicin, etoposide, 5-FU	p53-KO HCT-116 CRC	Increased MYB
Doxorubicin, etoposide, cytarabine, mafosfamide	HT1080 fibrosarcoma	Increased MYBL2
Temozolomide	Primary glioblastoma	ZEB1/miR-200c/MYB axis, increased MGMT and DNA repair
Cytarabine	AML	Upregulated miR-107 suppressed MYB
Olaparib	A2780 and SKOV3 OC	High MYBL2 levels increased CDCA8
Olaparib	AR-positive and -negative prostate cancer	MYB induction upon androgen deprivation, increased DNA damage response via MYB-TopBP1-ATR-CHK1
Bleomycin	NT2/D1 germ cell tumor	Ape1/ref-1 activates MYB DNA binding, increased Ape1/ref-1 stimulated DNA base excision repair
Chlorin e6	SW480 CRC	MYBL2 knockdown activated NF-κB and ABCG2

CRC: Colorectal carcinoma; ALL: acute lymphoblastic leukemia; BCL2: B-cell lymphoma 2; IGF2BP1: insulin-like growth factor 2 mRNA-binding protein 1; OS: osteosarcoma; NOX1: NADPH oxidase 1; MAPK: mitogen-activated protein kinase; NF-κB: nuclear factor k-light-chain-enhancer of activated B cells; 5-FU: 5-fluorouracil; ZEB1: zinc finger E-box-binding homeobox 1; MGMT: O-6-methylguanine DNA methyltransferase; AML: acute myeloid leukemia; OC: ovarian carcinoma; CDCA8: cell division cycle-associated 8; AR: androgen receptor; TopBP1: topoisomerase 2-binding protein 1; ATR: ataxia telangiectasia and Rad3 related protein; CHK1: checkpoint kinase 1.

#### Resistance to microtubule-targeting drugs

The vinca alkaloid vincristine was isolated from Madagascar periwinkle (*Catharanthus roseus*) in 1961 and approved for the therapy of leukemia in 1963. Vincristine interacts with tubulin and disrupts microtubules and is applied in combination with other drugs (e.g., as part of the CHOP therapy in combination with cyclophosphamide, doxorubicin, and prednisone/prednisolone)^[82]^. Upregulated MYB expression in p53-knockout CRC cells led to multidrug resistance, including resistance to vincristine^[54]^. In contrast, gene expression analysis of vincristine-resistant erythroleukemia cells revealed downregulation of MYBL1 compared with sensitive cells. Markedly, MYBL1 was the only downregulated gene from 58 studied genes showing different expression patterns in the resistant cells^[83]^. It nevertheless remained unclear to what extent MYBL1 suppression contributed to vincristine resistance.

The taxanes paclitaxel and docetaxel are antimitotics that stabilize microtubules. Paclitaxel was approved in 1984 for the therapy of advanced OC, whereas taxanes are applied for the treatment of various solid tumors now. The mechanisms leading to taxane resistance in various cancers were recently summarized^[84]^. The circular RNA circ_0004087 is overexpressed in prostate cancer cells and patient samples. It conveyed docetaxel resistance by binding to the cofactor *Staphylococcus* nuclease domain-containing protein 1 (SND1), which upregulated MYB transactivation followed by increased expression of the serine/threonine kinase budding uninhibited by benzimidazoles 1 (BUB1) and activation of the cellular mitosis error correction mechanism^[85]^. In addition, MYBL2 belongs to the five key microtubule-associated genes (MAGs) in lung adenocarcinomas. MAG upregulation indicated sensitivity to tubulin-targeting anticancer drugs such as paclitaxel and the vinca alkaloid vinblastine, as well as to cisplatin (for the latter see also Table 1)^[86]^. Taken together, studies on the role of MYB proteins on cancer resistance to tubulin-targeting drugs are scarce and under-represented. Given the importance of this drug class as anticancer agents, the currently existing knowledge warrants more in-depth studies on this matter. Table 3 summarizes the MYB-mediated resistance mechanisms to microtubule-targeting drugs.

**Table 3:** Cancer resistance to microtubule-targeting drugs mediated by MYB proteins

**Drug**	**Cancer/cell line**	**Resistance mechanism**
Vincristine	p53-knockout CRC cells	Upregulated MYB
Vincristine	K562-n/VCR erythroleukemia	Downregulation of MYBL1
Docetaxel	Prostate cancer cells and patient tissues	Overexpressed circ_0004087 binds SND1 and activates MYB, increased BUB1 and mitosis error correction mechanism
Paclitaxel, vinblastine	Lung adenocarcinoma patient samples	Low MYBL2

CRC: Colorectal carcinoma; SND1: *Staphylococcus* nuclease domain-containing protein 1; BUB1: budding uninhibited by benzimidazoles 1.

#### Resistance to nuclear receptor-targeting drugs and hormone therapy

Hormone therapy and specific drugs against nuclear receptors are of great importance for the treatment of various blood cancers and prostate, breast, and ovarian cancers. Retinoid receptor activating compounds retinoic acid (RA) and all-*trans* retinoic acid (ATRA) have been applied as curative agents for the therapy of acute promyelocytic leukemia (APL) since the 1980s based on their pronounced growth arrest and cell differentiation properties^[87]^. A correlation between retinoids and MYB proteins was discovered early. v-MYB-transformed BM2 chicken monoblasts were resistant to RA treatment, but the RA resistance was overcome by increased retinoid X receptor (RXR) expression^[88]^. The phosphatase inhibitor okadaic acid and overexpression of nuclear factor of activated T-cells 1 (NFAT1) induced sensitivity of v-MYB-transformed RA-resistant monoblasts to RA-mediated differentiation^[89,90]^. Knockdown of MYB-regulated IGF2BP1 enhanced differentiation of leukemia cells by ATRA^[59]^. In contrast, MYBL2 activity was upregulated via cyclin D1 suppression in neuroblastoma cells upon treatment with RA, which led to RA-mediated cell differentiation^[91]^.

Glucocorticoids (GCs, i.e., the steroids prednisone, prednisolone, and dexamethasone) activate GC receptors (GRs) to induce apoptosis, and they are widely applied for the therapy of blood cancers, e.g., as drugs of choice for the treatment of ALL. However, x resistance to GCs in ALL is associated with poor prognosis, which needs to be urgently addressed^[92]^. In pediatric patient-derived ALL xenografts, persistent MYB expression led to GC resistance by sustained expression of anti-apoptotic BCL2, which suppressed dexamethasone-mediated apoptosis^[93]^. Similar MYB- and BCL2-based anti-apoptotic effects were observed in dexamethasone-treated CTLL-2 cells^[94]^. In addition, MYBL2-transfected CTLL-2 cells were resistant to dexamethasone-mediated apoptosis^[62]^. Increased dexamethasone activity was observed upon suppression of MYB in ALL cells modified with lentiviral sh-c-Myb^[57]^. However, MYB was also required for GR promoter activation by dexamethasone-activated GR in ALL cells^[95]^. The MYC activator MYB was identified as a target of miR-103, which is a tumor suppressor downregulated in ALL. MYB-positive ALL cells with high miR-103 levels became highly sensitive to apoptosis induction by dexamethasone based on miR-103-mediated MYB and MYC suppression, while miR-103 inhibition led to reduced dexamethasone-mediated apoptosis^[96]^.

In contrast to agonistic retinoids and GCs, prominent anticancer therapies targeting estrogen and androgen receptors (ER and AR) aim at the suppression of ER and AR activities because of their tumor-promoting properties. Notably, MYB is an effector of ER signaling in ER-positive breast cancers^[97]^. The development of the triphenylethylene-based drug tamoxifen as an inhibitor/modulator of ER [selective estrogen-receptor modulator (SERM)] in the 1960s marked enormous progress in the therapy of breast cancer patients, but this progress was also accompanied by the discovery of tamoxifen-resistant or -insensitive tumors^[98]^. MCF-7 breast cancer cells are sensitive to tamoxifen; however, ongoing dose-escalating exposure to tamoxifen led to resistant TAM-MCF-7 cells. This tamoxifen resistance was characterized by high MYB expression and suppression of miR-200 (miR-200b and miR-200c), leading to upregulation of the EMT markers vimentin and ZEB1^[99]^. Similar resistance mechanisms were also described for DNA-targeting drugs (see above). In addition, cyclin D1 was overexpressed in tamoxifen-resistant breast cancer cells with upregulated MYB^[100]^. Moreover, MYBL2 overexpression was observed in tamoxifen-resistant breast cancer cells associated with upregulation of BIRC5 (survival), hyaluronan-mediated motility receptor (HMMR) (metastasis), as well as PLK1 and protein regulator of cytokinesis 1 (PRC1) (proliferation), while suppression of MYBL2 re-sensitized cells to tamoxifen^[101]^. Since tamoxifen also possesses partial ER agonistic activity, the steroidal drug fulvestrant was developed as a selective estrogen receptor degrader with activity against ER-positive tamoxifen-resistant breast cancer^[102]^. The investigation of the gene expression profile of fulvestrant-resistant breast cancer cells induced by zinc finger transcription factors revealed four gene clusters that overlapped with MYB-regulated genes, indicating a vital role of MYB in fulvestrant resistance^[103]^. Metabolism of fulvestrant significantly affects its anticancer activities. Glucuronidation of fulvestrant and the aromatase inhibitor anastrozole occurs via the UDP-glucuronosyltransferase 1A4 (UGT1A4), which was upregulated by fulvestrant in breast cancer cells via MYB. In addition, fulvestrant-mediated upregulation of *UGT1A4* mRNA was associated with the expression of *MRP1* mRNA, which encodes an important glucuronide export pump^[104]^. The enforced drug metabolism is an uncommon but notable MYB effect that deserves more attention in future resistance studies with other anticancer drugs prone to intracellular metabolism.

The activation of ARs by androgens (testosterone, dihydrotestosterone) drives prostate cancer. Androgen deprivation therapy is commonly applied; however, it is hampered by the appearance of resistant and refractory prostate tumors. MYB is upregulated in hormone-refractory prostate cancers^[105]^. The treatment of androgen-dependent prostate cancer cells with the antiandrogen enzalutamide also increased MYB expression, while a synthetic androgen (R1881) suppressed MYB^[66]^. The increased MYB expression upon enzalutamide treatment points to compensation of AR loss that mediates enzalutamide resistance^[106]^. CRPC cells are characterized by high MYB and MYBL2 levels, which are associated with aggressive and resistant phenotypes^[107,108]^. MYB interacts directly with AR, leading to ligand-independent AR activation and the establishment of problematic CRPC^[109]^. The combination of the semi-synthetic steroidal cytochrome P450 17A1 (CYP17A1) inhibitor abiraterone with prednisone is a first-line therapy of CRPC^[110]^. Abiraterone-sensitive prostate cancer cells revealed much higher MYB levels than abiraterone-resistant cells, and the reduction of MYB signaling seemed responsible for the development of abiraterone resistance^[111]^. The observations that antiandrogen therapy upregulates MYB expression and abiraterone activity depends on active MYB signaling might solve clinical problems of current CRPC therapy. Table 4 summarizes the MYB-mediated resistance mechanisms to hormone receptor-targeting drugs.

**Table 4:** Cancer resistance to hormone receptor-targeting drugs mediated by MYB proteins

**Drug**	**Cancer/cell line**	**Resistance mechanism**
Retinoic acid	v-MYB-transformed BM2 chicken monoblasts	v-MYB expression
ATRA	Leukemia cells	MYB-regulated IGF2BP1
Dexamethasone	Pediatric patient-derived ALL xenografts	MYB expression increased BCL2
Dexamethasone	CTLL-2	MYB expression increased BCL2, MYBL2 expression
Dexamethasone	697 pre B-cell ALL	MYB expression
Dexamethasone	CEM-C7H2 ALL	High MYB and MYC expression by miR-103 suppression
Tamoxifen	TAM-MCF-7 breast cancer	High MYB expression and suppressed miR-200b and miR-200c, upregulation of vimentin and ZEB1
Tamoxifen	MCF7/TamR and T47D/TamR breast cancer	MYBL2 overexpression and upregulation of BIRC5, HMMR, PLK1 and PRC1
Fulvestrant	Fulvestrant-resistant MCF-7 breast cancer	Gene clusters overlapping with MYB-regulated genes
Fulvestrant	MCF-7 breast cancer	MYB-mediated glucuronidation by UGT1A4, MRP1 upregulation
Enzalutamide	AR-sensitive VCaP and LNCaP prostate cancer	Increased MYB compensates AR loss
Abiraterone	LNCaP-AR and 22Rv1-AR prostate cancer	Low MYB

ATRA: All-*trans* retinoic acid; IGF2BP1: insulin-like growth factor 2 mRNA-binding protein 1; ALL: acute lymphoblastic leukemia; BCL2: B-cell lymphoma 2; ZEB1: zinc finger E-box-binding homeobox 1; HMMR: hyaluronan-mediated motility receptor; PLK1: polo-like kinase 1; PRC1: protein regulator of cytokinesis 1; UGT1A4: UDP-glucuronosyltransferase 1A4; AR: androgen receptor.

### Resistance to targeted therapy, epigenetic drugs and immune therapy

Conventional chemotherapy was complemented by the progress in the development of targeted anticancer drugs (e.g., protein kinase inhibitors and monoclonal antibodies) over the last 20 years, which led to new therapeutic options^[112]^. Similarly, epigenetic drugs and immune therapeutics have become indispensable components of the current arsenal of drugs^[113,114]^. However, drug resistance formation and insensitive cancers warrant a closer look at mechanisms mediated by MYB proteins.

#### Resistance to targeted therapy

Several small-molecule protein kinase inhibitors and monoclonal anti-kinase/anti-growth factor antibodies are now applied for targeted cancer therapy, and numerous promising new inhibitor molecules are currently in clinical trials. The quinazoline-based EGFR inhibitor erlotinib is used for the treatment of non-small cell lung cancer (NSCLC) and metastatic pancreatic cancer^[115]^. The upregulation of MYBL2 as a key MAG in lung adenocarcinomas was associated with low response to erlotinib^[86]^. The monoclonal anti-EGFR antibody cetuximab is approved for the therapy of metastatic CRC and head-and-neck squamous cell carcinoma^[116]^. MYB was strongly downregulated in cetuximab-resistant CRC cells, while MYB expression was associated with cetuximab sensitivity and increased tumor immune cell infiltration^[117]^. In contrast to that, upregulated MYBL2 was correlated with cetuximab resistance in NSCLC cells, which was mediated by Src-family kinase Yes- and Lin-promoted EGFR nuclear translocation and the binding of nuclear EGFR complexes to the MYBL2 promoter^[118]^. Together with the data on erlotinib, MYBL2 expression appears to have a negative effect on the activity of EGFR inhibitors. The antibody trastuzumab inhibits human epidermal growth factor receptor 2 (HER2/ErbB2) in HER2-positive breast cancer and was one of the first approved targeted therapies in the late 1990s^[119]^. The expression of various miRNAs was modulated (e.g., miR-34c-3p and miR-195-5p were downregulated) in trastuzumab-resistant breast cancer cells and in the plasma of trastuzumab-resistant cancer patients, which led to significantly increased MYB levels^[120]^.

The urethane-based multi-kinase inhibitor sorafenib was initially developed as an inhibitor of the serine/threonine kinase rapidly accelerated fibrosarcoma (Raf), but sorafenib turned out to be a potent inhibitor of various receptor tyrosine kinases (RTKs) such as vascular endothelial growth factor receptor (VEGFR), platelet-derived growth factor receptor (PDGFR), and c-KIT. The drug is approved for the therapy of advanced renal cell carcinoma (RCC) and hepatocellular carcinoma (HCC)^[121]^. Notably, high MYBL1 levels were associated with poor prognosis in HCC patients, and MYBL1 was identified as a promotor of tumor angiogenesis and sorafenib resistance in HCC cells stably expressing MYBL1 (HepG2-MYBL1 cells) by activation of angiopoietin 2 (ANGPT2). The induction of ANGPT2 expression by MYBL1 required the cofactors protein arginine methyltransferase 5 (PRMT5), methylosome protein 50 (MEP50), and WD repeat-containing protein 5 (WDR5)^[122]^. Notably, the inhibition of MYBL2 phosphorylation by VEGFR/PDGFR inhibitors can promote tumor growth and survival in VHL disease, which is relevant since VEGFR inhibitors are applied for VHL disease therapy. The 2-oxindole-based sunitinib analog and VEGFR/PDGFR inhibitor SU4312 prevented Tyr15-phosphorylation of MYBL2, leading to proteasomal degradation of MYBL2 and cell proliferation. MYBL2 degradation was mediated by interaction with the ubiquitin ligase pVHL, which was identified as another mechanism of pVHL in addition to HIF-1α degradation. Thus, VEGFR inhibitor therapy might have limited efficacy in VHL patients with functional pVHL. However, MYBL2 was also degraded, although more slowly, in pVHL-deficient 786-O renal RCC cells, indicating further ubiquitin ligases involved in this process^[123]^. Indeed, there is evidence that MYB is targeted by other ubiquitin ligases such as the F-box protein Fbxw7 upon MYB-phosphorylation by Nemo-like kinase (NLK)^[124]^.

The development of the pyridin-3-ylpyrimidine-based kinase inhibitor imatinib (inhibitor of ABL, PDGFR and c-KIT) was a milestone for the therapy of chronic myeloid leukemia (CML) with breakpoint cluster region protein (BCR)/ABL fusion proteins (generated by the Philadelphia chromosome, Ph^+^), and also became an important drug for the treatment of gastrointestinal stromal tumor based on its c-KIT inhibitory activity^[125]^. Imatinib-resistant BCR/ABL-positive KCL22/SR CML cells exhibited suppressed MYB levels in contrast to cells of the sensitive parent cell line^[126]^. However, MYB has a longer PI3K-signaling-dependent half-life in BCR/ABL-expressing cells than in normal cells, and the presence of degradation-resistant MYB mutants in transfected CML cells conveyed resistance to imatinib-mediated apoptosis independent from the MYB expression level^[127]^. The anti-apoptotic effect of the degradation-resistant MYB mutant was enhanced by co-expression of BCL2^[128]^. The Kaiso transcription factor was expressed in imatinib-sensitive CML cells and downregulated in imatinib-resistant cells. Deletion of Kaiso increased MYB expression and promoted cell survival and proliferation^[129]^. MYB was approximately two-fold upregulated in imatinib-resistant BCR/ABL1-positive KCL-22R CML cells compared with sensitive cells as part of an oncogenic myeloid differentiation-blocking network comprising MYC/miR-150/MYB/miR-155/PU-1. MYC reduced miR-150, leading to upregulation of MYB, which in turn downregulated miR-155 in KCL-22R cells. In contrast to that, MYB levels did not differ between naive K562 and resistant K562R CML cells^[130]^. However, these results are in stark contrast to the observed 11-fold suppression of MYB in imatinib-resistant KCL22/SR cells, which were obtained from KCL22 cells analogously to the KCL-22R cells^[125]^. It is possible that different clones were isolated from KCL22 parent cells in these two different studies. A pediatric ALL patient survey including BCR/ABL1 patients who received imatinib therapy, identified enhanced MYB regulation by alternative MYB promoters in relapsed/therapy-resistant patients, which was accompanied by enhanced KRAS signaling, increased ABC transporter expression (ABCA2, ABCB5, ABCC10) and elevated drug degradation enzyme levels (CYP1A2, CYP2C9, CYP3A5)^[131]^.

The pyrimidine-based Janus kinase (JAK) inhibitor cerdulatinib is in clinical development for the therapy of relapsed/refractory T-cell lymphoma^[132]^. The MYB-tyrosin kinase 2 (TYK2) fusion protein induced cerdulatinib resistance in B-ALL cells by upregulation of JAK/STAT signaling and JAK1 overexpression^[133]^. Clinical trials of the investigational pyrazole-based CHK1 inhibitor prexasertib exhibited considerable anticancer activity against recurrent OC and advanced squamous cell carcinoma^[134]^. A preclinical study using NSCLC cells revealed prexasertib resistance upon MYBL2 deletion by CRISPR-Cas9 methods. The premature mitosis-promoting MMB-forkhead box protein M1 (FOXM1) complex was required for sensitivity to prexasertib and the induction of replication catastrophe by this CHK1 inhibitor^[135]^. Table 5 summarizes the MYB-mediated resistance mechanisms to targeted drugs.

**Table 5:** Cancer resistance to targeted drugs mediated by MYB proteins

**Drug**	**Cancer/cell line**	**Resistance mechanism**
Erlotinib	Lung adenocarcinoma patient samples	Upregulation of key MAG MYBL2
Cetuximab	CACO2-CR CRC	Suppressed MYB
Cetuximab	NCI-H226 NSCLC	Upregulated MYBL2 via Src-family kinase Yes- and Lin-promoted EGFR nuclear translocation
Trastuzumab	BT-474 breast cancer, plasma of resistant patients	Increased MYB by suppression of miR-34c-3p and miR-195-5p
Sorafenib	HepG2-MYBL1 HCC	MYBL1 promoted angiogenesis and resistance by activation of ANGPT2
SU4312	293T and 786-O (RCC) cells transfected with 3XFLAG-pVHL	Dephosphorylated MYBL2 prone to ubiquitination and proteasomal degradation by pVHL
Imatinib	BCR/ABL-positive KCL22/SR CML	Suppressed MYB
Imatinib	Transfected K562 CML	PI3K-dependent degradation-resistant MYB mutants
Imatinib	Imatinib-resistant cells derived from K562 CML	Downregulated Kaiso increased MYB
Imatinib	KCL-22R CML	Increased MYB, MYC/miR-150/MYB/miR-155/PU-1 axis
Imatinib and others	ALL patients	Enhanced MYB regulation by alternative MYB promoters
Cerdulatinib	B-ALL cells	MYB-TYK2 fusion protein, upregulation of JAK/STAT signaling
Prexasertib	A549 and H460 NSCLC	MYBL2 deletion by CRISPR-Cas9, inhibition of MMB-FOXM1 complex and replication catastrophe

MAG: Microtubule-associated gene; CRC: colorectal carcinoma; NSCLC: non-small cell lung cancer; EGFR: epidermal growth factor receptor; HCC: hepatocellular carcinoma; ANGPT2: activation of angiopoietin 2; RCC: renal cell carcinoma; pVHL: protein of the von Hippel-Lindau; BCR: breakpoint cluster region protein; CML: chronic myeloid leukemia; PI3K: phosphoinositide 3 kinase; ALL: acute lymphoblastic leukemia; TYK2: tyrosin kinase 2; JAK: Janus kinase; STAT: signal transduction activator of transcription; MMB: MYBL2-MuvB; FOXM1: forkhead box protein M1.

#### Resistance to epigenetic drugs and immune therapy

The prolific field of cancer epigenetics has led to the development of several new and promising anticancer drugs such as DNA demethylase inhibitors or histone deacetylase (HDAC) inhibitors^[113]^. MYB was highly sensitive to sustained histone acetylation by the natural HDAC inhibitor trichostatin A (TSA) in leukemia cells^[136]^. Conversely, MYB proteins are able to regulate HDAC inhibitor activity. Sodium butyrate is a structurally simple differentiation-inducing agent with HDAC inhibitory activity. In MCF-7 breast cancer cells, knockdown of MYB led to inactivation of its anti-apoptotic target BCL2, followed by increased sensitivity to sodium butyrate-induced apoptosis^[137]^. Vorinostat [also known as suberoyl anilide hydroxamic acid (SAHA)] was the first HDAC inhibitor approved for cancer therapy^[138]^. Acute T-cell leukemia cells sensitive to vorinostat-induced cell death showed MYBL2 downregulation upon treatment, while resistant cells expressing the CDK inhibitor p16^INK4A^ lost the ability to suppress MYBL2^[139]^. Knockdown of MYB in myeloid leukemia cells induced pro-apoptotic Bcl-2 related ovarian killer (BOK) and Bcl-2 interacting mediator of cell death (BIM) and led to sensitization to panobinostat, which is another clinically approved HDAC inhibitor. In addition, mice bearing myeloid leukemia cells with inducible MYB shRNA showed prolonged survival when panobinostat was combined with MYB knockdown^[140]^. Thus, loss of MYBL2-mediated cell cycle regulation and promotion of anti-apoptotic mechanisms by MYB correlate with HDAC inhibitor resistance.

**Figure 1:**
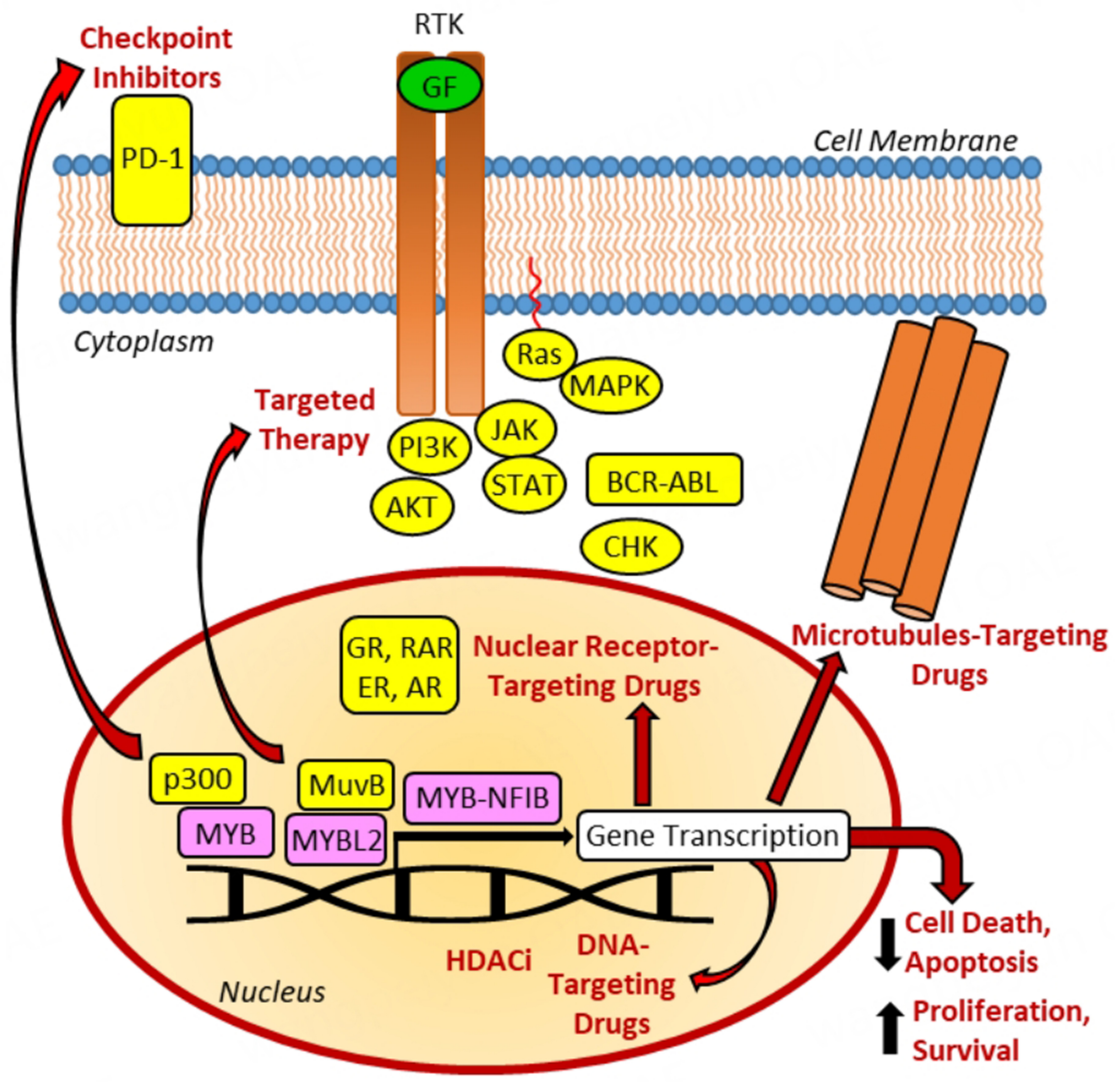
Gene transcription regulated by MYB, MYBL2, and MYB fusions (e.g., MYB-NFIB) plays a crucial role in the resistance to DNA-targeting, microtubule-targeting and nuclear receptor-targeting drugs, as well as to targeted therapy, HDACi, and checkpoint inhibitors. MYB proteins were associated with suppressed cell death and promotion of cell proliferation and survival. Resistance to DNA-targeting drugs includes platinum complexes, topoisomerase inhibitors, antimetabolites, PARP inhibitors, alkylating and photodynamic drugs. Microtubule-targeting drugs include vinca alkaloids and taxanes. Resistance to nuclear receptors refers to activators of GR and RAR, as well as inhibitors of AR and ER. Resistance to targeted therapy includes inhibitors of RTKs with implications for oncogenic Ras-MAPK and PI3K-AKT signaling, inhibitors of JAK-STAT signaling, as well as inhibitors of BCR/ABL tyrosine kinase and CHK. Resistance to epigenetic drugs and checkpoint inhibitors includes HDACi and anti-PD-1 antibodies. NFIB: Nuclear factor IB; HDACi: histone deacetylase inhibitors; PARP: phthalazinone-based poly (ADP-ribose) polymerase; GR: glucocorticoid receptor; RAR: retinoic acid receptor; AR: androgen receptors; ER: estrogen receptors; RTKs: receptor tyrosine kinases; MAPK: mitogen-activated protein kinase; PI3K: phosphoinositide 3 kinase; AKT: protein kinase B; JAK: Janus kinase; STAT: signal transduction activator of transcription; BCR: breakpoint cluster region protein; CHK: checkpoint kinase; PD-1: programmed death 1.

High responses to monoclonal antibodies targeting the immune checkpoints cytotoxic T-lymphocyte-associated protein 4 (CTLA-4) (e.g., ipilimumab) and programmed death 1 (PD-1) (e.g., pembrolizumab and nivolumab) led to the approval of these checkpoint inhibitors for the treatment of various cancers^[141]^. In an *in vivo* CRC model, the expression of MYB upregulated the Hsp70 isoform GRP78 of the endoplasmic reticulum, leading to unfolded protein response (UPR) and reduced activation of tumor-infiltrating CD8^+^ T-cells. These MYB-correlated effects were responsible for resistance of CRC to anti-PD-1 antibody treatment^[142]^. Murine ID8 OC was resistant to anti-PD-1 antibody (BE0273) *in vivo* treatment due to MYBL2-mediated upregulation of C-C-motif chemokine ligand 2 (CCL2) followed by enhanced recruitment of immunosuppressive macrophages; however, suppression of MYBL2 by shMYBL2 sensitized these tumors to anti-PD-1 treatment by reducing tumor-associated macrophage (TAM) numbers^[143]^. Both studies underline the relevance of MYB and MYBL2 for anti-PD-1 therapy resistance. Checkpoint inhibitors are a relatively new class of anticancer drugs compared with other well-established drug classes described in this work. Nevertheless, the growing number of checkpoint inhibitors in clinical development and therapy will likely increase the knowledge of the roles of MYB transcription factors in immune therapy resistance in the future. Table 6 summarizes the MYB-mediated resistance mechanisms to epigenetic drugs and immune therapeutics. Figure 1 provides an overview of MYB-mediated cancer drug resistance and affected drug compound classes.

**Table 6:** Cancer resistance to epigenetic drugs and immune therapeutics mediated by MYB proteins

**Drug**	**Cancer/cell line**	**Resistance mechanism**
Butyrate	MCF-7 breast cancer	Activated BCL2 by MYB
Vorinostat	CEM acute T-cell leukemia	Cells expressing p16^INK4A^ lost MYBL2 suppression ability
Panobinostat	U937 and K562 myeloid leukemia	MYB suppression of BOK and BIM
Anti-PD-1 antibody	CT26 CRC	MYB upregulated GRP78 and UPR, reduced activation of CD8^+^ T-cells
Anti-PD-1 antibody BE0273	ID8 OC	MYBL2-mediated upregulation of CCL2 and enhanced recruitment of immunosuppressive macrophages

BCL2: B-cell lymphoma 2; BOK: Bcl-2 related ovarian killer; BIM: Bcl-2 interacting mediator of cell death; CRC: colorectal carcinoma; UPR: unfolded protein response; OC: ovarian carcinoma.

### Drugs targeting MYB proteins as cancer therapeutics

Based on the relevance of MYB proteins for drug resistance, compounds suppressing MYB function have the potential to become suitable anticancer drugs. Disruption of MYB interaction with cofactors and promotion of post-translational MYB inactivation and degradation are the main strategies in MYB-directed drug development. Although a focus is set on the structure and function of small-molecule MYB regulators, MYB-targeting vaccines, polypeptides and antisense oligonucleotides are also described and discussed.

#### Oligonucleotides and vaccines

The first MYB inhibitors were MYB antisense oligodeoxynucleotides (ODNs), which bound to MYB mRNA. The proliferation of MYC-expressing HL-60 leukemia and MYB-expressing CRC cells (LoVo, doxorubicin-resistant LoVo/Dx, and COLO-205) was inhibited by ODNs^[57,144,145]^. In addition, the combination of MYB antisense phosphorothioate ODNs with cisplatin was active against LoVo/Dx CRC tumors *in vitro* and *in vivo*^[146]^. Antisense drugs targeting non-coding RNAs and miRNA-based drugs are also promising strategies to suppress MYB in cancers^[147]^. In terms of immune therapy, the treatment with the MYB-targeting TetMYB DNA vaccine showed prophylactic effects in adenomatous polyposis mouse models and overcame anti-PD-1 resistance in MYB-expressing CT26 and MC38 CRC models by increased CD8^+^ T-cell activation^[148,149]^. A clinical phase 1 trial with the TetMYB vaccine in combination with the PD-1 inhibitor tislelizumab was launched for CRC and ACC supported by a reported clinical response of MYB-NFIB-positive patients to the PD-1 inhibitor pembrolizumab^[150,151]^.

#### Kinase inhibitors

**Figure 2:**
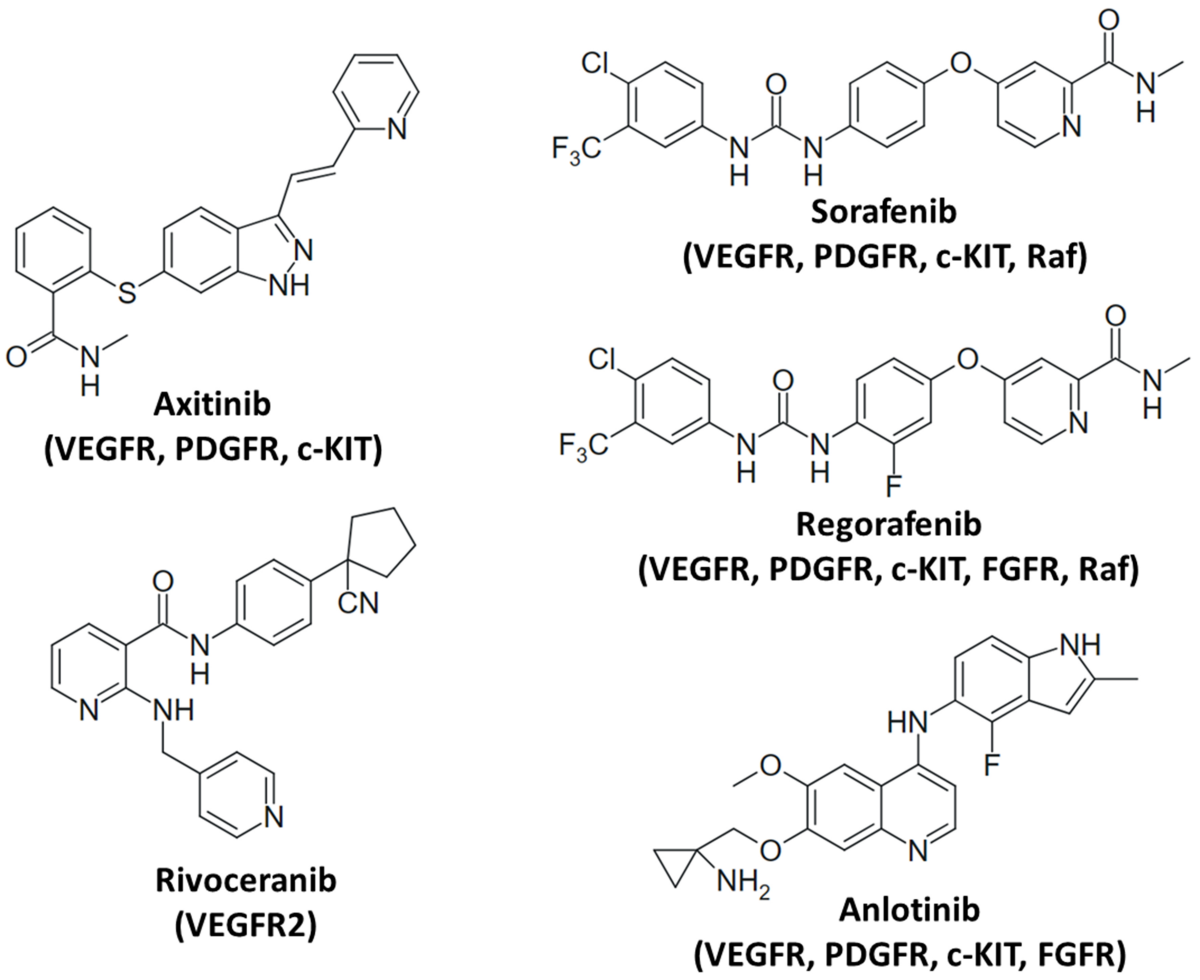
Structures of VEGFR-targeting (multi-)kinase inhibitors (targets in brackets) with effects on MYB activity and/or MYB-dependent cancers. VEGFR: Vascular endothelial growth factor receptor.

Several multi-kinase inhibitors were clinically studied in aggressive and therapy-resistant cancers with altered MYB (e.g., ACC) and revealed controversial outcomes^[152]^. A study with dovitinib showed no correlation between MYB status and patient response, while the highest responses to lenvatinib were found in ACC patients with low or absent MYB^[153,154]^. Nevertheless, prolonged progression-free survival (PFS) upon axitinib treatment was observed in MYB-NFIB-positive patients^[155]^. Sorafenib also exhibited considerable responses and prolonged stable disease in ACC patients^[156,157]^. The combination of anlotinib with the tubulin binder eribulin showed an enduring response in a MYB-NFIB and BCOR (BCL6 corepressor) mutant ACC patient after chemotherapy (carboplatin, cyclophosphamide, doxorubicin) had failed^[158]^. A phase 2 trial of anlotinib in metastatic ACC revealed activity and controllable toxicity, indicating a possible application as palliative therapy of advanced ACC^[159]^. The multi-kinase inhibitors anlotinib, axitinib, and sorafenib block angiogenesis by targeting VEGFRs, and angiogenesis is an important process in ACC development^[30]^. The selective VEGFR2 inhibitor rivoceranib also showed promising anticancer activity in a phase 2 trial with recurrent or metastatic ACC^[160]^. It seems that ACC patients lacking MYB-NFIB also benefit from antiangiogenic therapy. However, patients with MYB-NFIB expressing ACC showed increased tumor vascularization and VEGF production compared with non-expressing patients, indicating a considerable pro-angiogenic effect of MYB-NFIB in ACC^[161]^. At the preclinical stage, the sorafenib analog regorafenib was active against MYB-translocated patient-derived ACC6 and ACC11 cells and inhibited ACC growth in mouse and zebrafish models^[162]^. Although the roles of MYB and MYB-NFIB in the formation of recurrent ACC after treatment are not completely understood, the observed activities of certain VEGFR-targeting kinase inhibitors are promising. The structures of VEGFR inhibitors with activity against ACC are shown in Figure 2.

**Figure 3:**
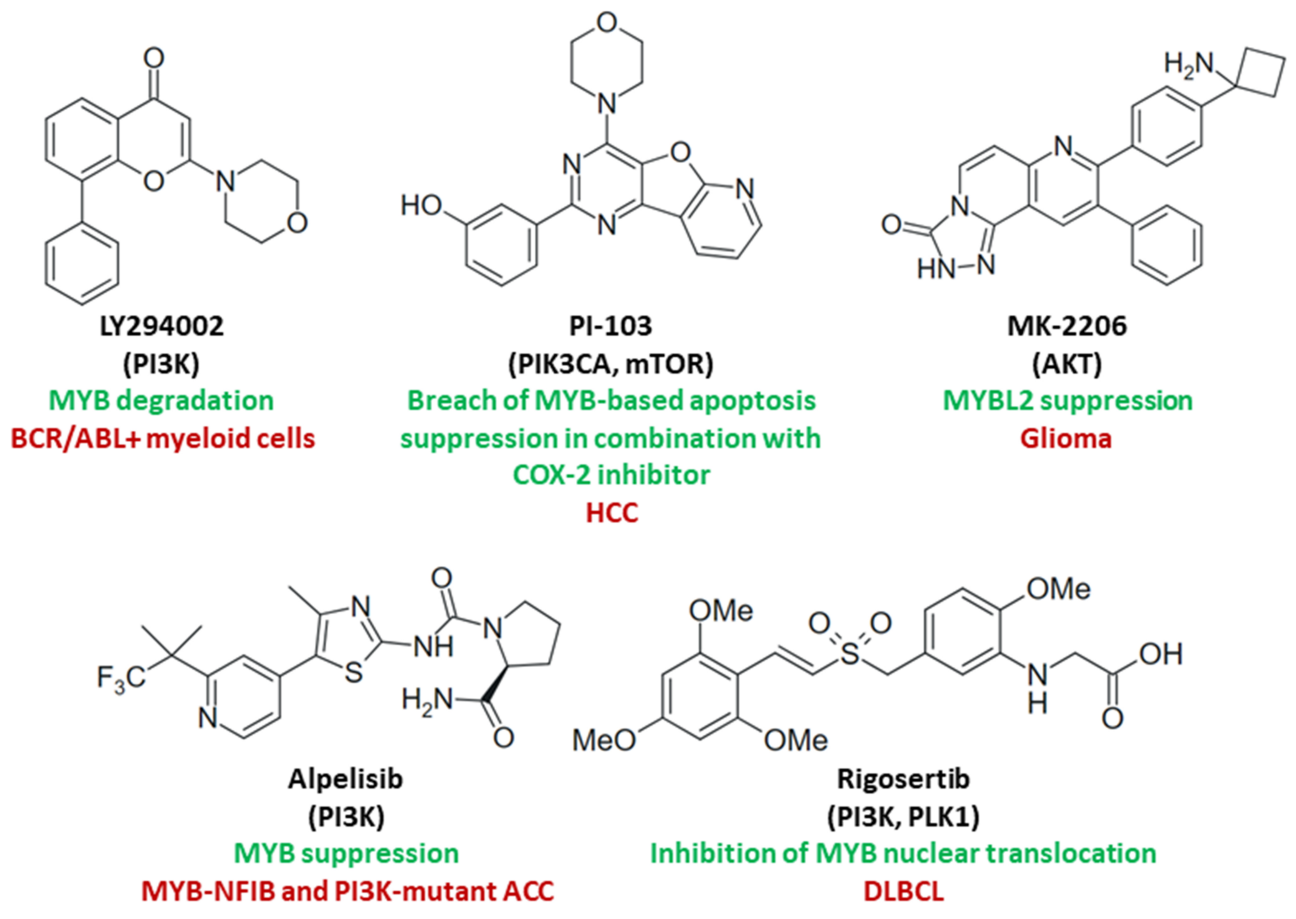
Structures of inhibitors of PI3K-AKT-mTOR signaling (kinase targets in brackets) with effects on MYB activity and/or MYB-dependent cancers (green: mechanisms; red: targeted cancers). PI3K: Phosphoinositide 3 kinase; AKT: protein kinase B; mTOR: mammalian target of rapamycin.

The PI3K inhibitor LY294002 downregulated MYB in parent and BCR/ABL-positive murine IL3-dependent 32Dcl3 myeloid precursor cells by reducing MYB stability and promoting MYB degradation, which has the potential to overcome imatinib resistance^[127]^. The cytotoxicity of the Ras-mimetic PI3K/PLK1 inhibitor rigosertib in diffuse large B-cell lymphoma (DLBCL) cells was associated with MYB suppression and inhibition of nuclear MYB translocation by sumoylation. Because nuclear MYB expression is associated with poor prognosis in DLBCL, rigosertib has the potential to become a suitable therapy for this imperiled DLBCL patient sub-group^[163]^. In MYB-NFIB-positive and PI3KC-amplified patient-derived ACC, the PI3K inhibitor alpelisib increased the activity of cisplatin *in vivo* by suppression of MYB levels; however, stronger tumor growth inhibition and MYB downregulation were observed in MYB-NFIB and PIK3CA^R88Q^ constitutively active mutant ACC in combination with RA^[164]^. The suppression of apoptosis in HCC cells depended on MYC and E2F1, which upregulated PI3K signaling as well as MYB and its downstream target cyclooxygenase 2 (COX-2). The combination of the COX-2 inhibitor CAY10404 with the dual PI3K/mammalian target of rapamycin (mTOR) inhibitor PI-103 circumvented the anti-apoptotic mechanisms and showed high antiproliferative and apoptosis-inducing effects on Huh1, Huh7, and Alexander HCC cells^[165]^. The oncogenic connection of MYB with COX-2 was also described for inflammation-driven breast cancers, which might be sensitive to a combined PIK3 inhibitor and COX-2 inhibitor therapy^[166]^. Thus, inhibitors of PI3K/AKT/mTOR signaling are promising drug candidates for the treatment of MYB-dependent cancers, which is supported by the outcome of clinical studies with the mTOR inhibitor everolimus for the therapy of advanced ACC, revealing promising activity as monotherapy or in combination with the immune-modulating and antiangiogenic drug lenalidomide^[167,168]^. MYBL2 induction by AKT/FOXM1 signaling was associated with glioma progression and anti-apoptotic mechanisms; however, the AKT inhibitor MK-2206 strongly suppressed MYBL2 in U251 glioma cells and can be a useful drug to tackle the anti-apoptotic mechanisms in glioma based on the AKT-FOXM-MYBL2 axis^[169]^. Structures of the described inhibitors of PI3K-AKT-mTOR signaling are shown in Figure 3.

**Figure 4:**
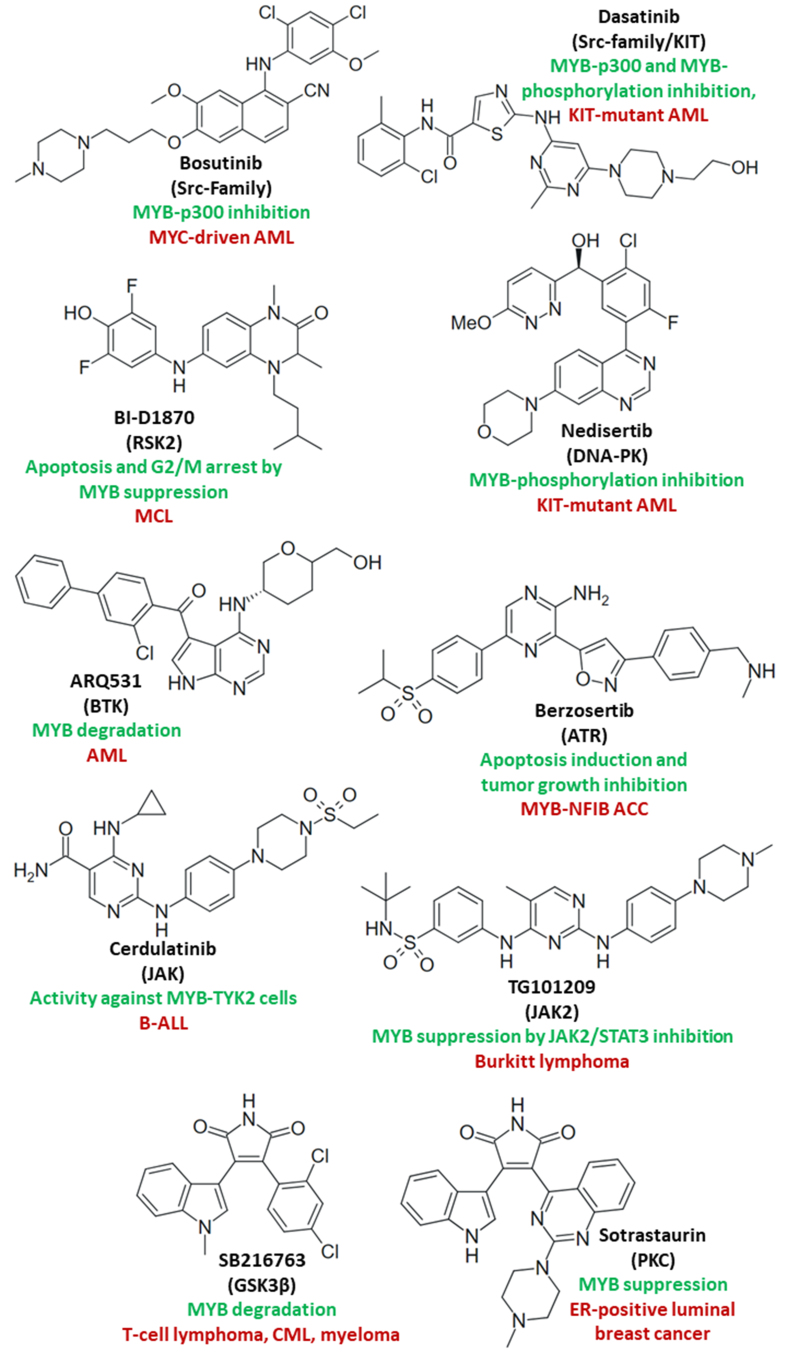
Structures of miscellaneous protein kinase inhibitors (kinase targets in brackets) with effects on MYB activity and/or MYB-dependent cancers (green: mechanisms; red: targeted cancers).

Notably, Src-family protein kinase inhibitors such as bosutinib inhibited MYB by blocking its interaction with the coactivator p300, which is a new mechanism of action for these compounds. Bosutinib killed MYC-expressing HL-60 AML cells in a MYB-dependent way and induced HL-60 differentiation by CD11b expression. Dasatinib was also identified as a MYB inhibitor, albeit at a higher concentration than bosutinib^[170]^. MYB and MYC phosphorylation were synergistically suppressed in KIT-mutant FDC-P1 AML cells by the combination of the Src/KIT inhibitor dasatinib with the DNA-PK inhibitor nedisertib^[171]^. Incidentally, a phase 2 study of dasatinib for the treatment of KIT-positive recurrent and metastatic ACC revealed 50% stable disease and, therefore, the MYB-suppressing effects of Src-family/KIT inhibitors might be of relevance for ACC therapy too^[172]^. BI-D1870 is a specific inhibitor of the N-terminal kinase domain of the MAPK-downstream serine/threonine kinase ribosomal protein S6 kinase 2 (RSK2), and BI-D1870-mediated RSK2 inhibition led to suppression of MYB and MYC accompanied by G2/M cell cycle arrest and apoptosis induction in MCL/mantle cell lymphoma cells (Jeko-1, KPUM-YY1, MINO, and Z-138 cell lines). Notably, the combination of BI-D1870 with the BCL2 inhibitor venetoclax exhibited high activity against cells of MCL cell lines, which responded only moderately to venetoclax monotherapy. This provides a hint at the circumvention of anti-apoptotic resistance mechanisms by RSK2 inhibition and associated MYC/MYB downregulation^[173]^. The Bruton tyrosine kinase (BTK) inhibitor ARQ531 was 10-fold more active than the clinically approved BTK inhibitor ibrutinib against a panel of AML cell lines. The distinctly increased antileukemic activity of ARQ531 in comparison to ibrutinib was based on a combination of BTK inhibition and enforced proteasomal MYB degradation, which promoted apoptosis. The superior activity of ARQ531 compared to ibrutinib has the potential to prolong remission and delay relapse and resistance formation in AML patients^[174]^. TYK2 rearrangements are responsible for poor ALL prognosis and high-risk ALL. The JAK inhibitor cerdulatinib was active against MYB-TYK2 fusion B-cell ALL *in vitro* and *in vivo* and has the potential to become a possible treatment option for high-risk ALL patients with MYB-TYK2 fusion^[175]^. The selective JAK2 inhibitor TG101209 induced cell differentiation, apoptosis, and G2/M cell cycle arrest in Raji and Ramos Burkitt lymphoma cells by downregulation of MYB upon inhibition of JAK2/STAT3 signaling. TG101209 also sensitized Burkitt lymphoma cells to doxorubicin in a synergistic way and was active against Ramos xenografts *in vivo*^[176]^. ATR is a DNA-damage sensor serine/threonine kinase overexpressed in ACC since it is a downstream factor of MYB and MYB-NFIB signaling. MYB upregulates DNA repair, leading to ACC resistance to genotoxic stress. The ATR inhibitor berzosertib induced apoptosis in MYB-NFIB ACC1 and ACC2 cells and inhibited tumor growth of patient-derived ACC xenografts^[177]^. Thus, berzosertib inhibits a MYB-dependent target of the DNA repair machinery and might have great potential in combination with DNA-targeting anticancer drugs for ACC therapy. LiCl and SB216763, both inhibitors of the serine/threonine glycogen synthase kinase 3β (GSK3β), were antiproliferative against Jurkat (acute T-cell leukemia), K562 (CML), and RPMI-8226 (myeloma) cells by promotion of proteasomal MYB degradation and suppression of BCL2, survivin, and MYC. MYB and lymphoid enhancer-binding factor-1 (LEF-1) cooperated in the prevention of apoptosis in the leukemia cells, and GSK3β inhibition was able to circumvent this anti-apoptotic pathway^[178]^. The protein kinase C (PKC) inhibitor sotrastaurin was active against ER-positive T-47D luminal breast cancer cells by downregulation of the ER effector MYB, suggesting a promising role of MYB inhibition in the prevention and treatment of tamoxifen-resistant ER-positive breast cancers^[179]^. The structures of the described protein kinase inhibitors are shown in Figure 4.

**Figure 5:**
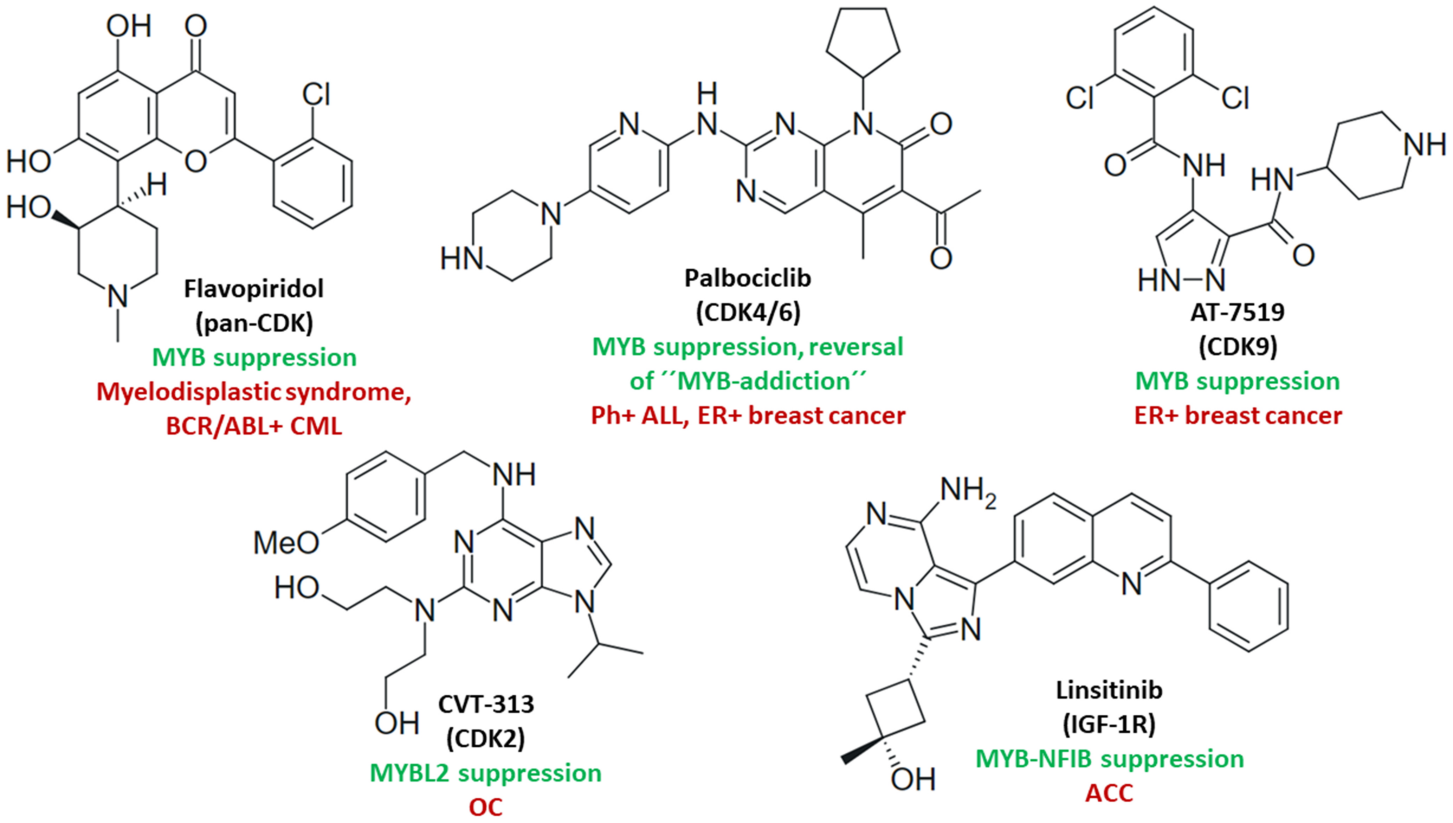
Structures of CDK inhibitors and the IGF-1R inhibitor linsitinib with effects on MYB activity and/or MYB-dependent cancers (green: mechanisms; red: targeted cancers). CDK: Cyclin-dependent kinase; IGF-1R: insulin-like growth factor 1 receptor.

MYB proteins play a vital role in cell cycle progression and various CDK inhibitors were investigated as MYB-interfering agents [Figure 5]. Flavopiridol was described as a MYB-downregulating CDK inhibitor that suppressed myelodysplastic syndrome and reduced myeloid cell numbers in MYB-hyperactivated zebrafish^[180]^. The combination of flavopiridol with imatinib showed enhanced efficacy against a MYB-expressing BCR/ABL1-positive CML zebrafish model as compared to imatinib monotherapy^[181]^. The “MYB addiction” of Ph^+^ ALL cells was mediated by CDK6 and BCL2. CDK4/6 inhibitor palbociclib plus BCL2 inhibitor venetoclax revealed marked effects on BV173 (synergistic) and SUP-B15 (additive) Ph^+^ ALL cells. In mice bearing Ph^+^ ALL-674 or ALL-1222 cells, palbociclib plus BCL2 inhibitor (sabutoclax) significantly decreased the peripheral leukemia load^[182]^. Thus, flavopiridol and palbociclib have the potential to prevent imatinib resistance formation in CML and ALL. The CDK9 inhibitors flavopiridol and AT7519 also showed high antiproliferative activity against MYB- and ER-positive breast carcinoma cells, along with induction of apoptosis and G2/M arrest upon suppression of MYB, BCL2, MCL-1, and CCNB1 (cyclin B1), while ER-negative MYB-negative cells were resistant to CDK9 inhibitors^[183]^. Analogously to the PKC inhibitor sotrastaurin, these CDK inhibitors can be useful for the management of ER-positive breast cancers developing tamoxifen resistance. CDK2 activated MYBL2 by phosphorylation, leading to anti-PD-1 resistance in OC, but the CDK2 inhibitor CVT-313 suppressed MYBL2 in A2780 and SKOV3 OC cells and circumvented anti-PD-1 resistance of murine ID8 OC by modulation of the tumor microenvironment^[143]^.

The IGF-1R is activated by binding to the growth factors IGF-1 and IGF-2 and overexpressed in various cancers^[184]^. AKT-dependent IGF-1R signaling and the IGF-2/IGF-1R/MYB-NFIB axis were identified as promising targets for ACC therapy^[185]^. The RTKs IGF-1R, EGFR, and MET were overexpressed in patient-derived ACC cells, while differentiation of ACC cells and synergistic inhibition of ACC xenograft growth was achieved by the combination of linsitinib (IGF-1R inhibitor, Figure 5), gefitinib (EGFR inhibitor), and crizotinib (MET inhibitor). However, only linsitinib was responsible for MYB-NFIB suppression, which was selective, given that wild-type/nonfused MYB expression was not affected^[186]^. Altered/overexpressed MYB was responsible for breast and colon cancer sensitivity to the anti-IGF-1R antibody figitumumab, and the combination with ErbB family inhibitors such as cetuximab, dacomitinib (both in colon cancers), or trastuzumab (in breast cancers), as well as combination with the CDK4/6 inhibitor palbociclib or oxaliplatin (both in colon cancers) led to synergistic effects^[187]^. A phase 1 trial of figitumumab combined with the EGFR/HER2/HER4 inhibitor dacomitinib revealed objective responses in advanced ACC, OC, and salivary gland cancer patients^[188]^. The potential of IGF-1R inhibitors to suppress MYB in ACC shows particular promise. ACC tumorigenesis and recurrence were associated with MYB and Notch signaling, and Notch ligand expression was upregulated by MYB, followed by activation of Notch in a paracrine way^[189]^. Notch signaling activates IGF-1R and the combination of the anti-IGF-1R antibody dalotuzumumab with the Notch inhibitor MK-0752 was well-tolerated by patients with advanced solid tumors^[190,191]^. Thus, established IGF-1R inhibitors in combination with Notch inhibitors that showed clinical activity against ACC (e.g., the pan-Notch inhibitor crenigacestat or the γ-secretase inhibitor AL101) can be a suitable option for future clinical trials with advanced/recurrent ACC^[30]^.

#### Epigenetic drugs

**Figure 6:**
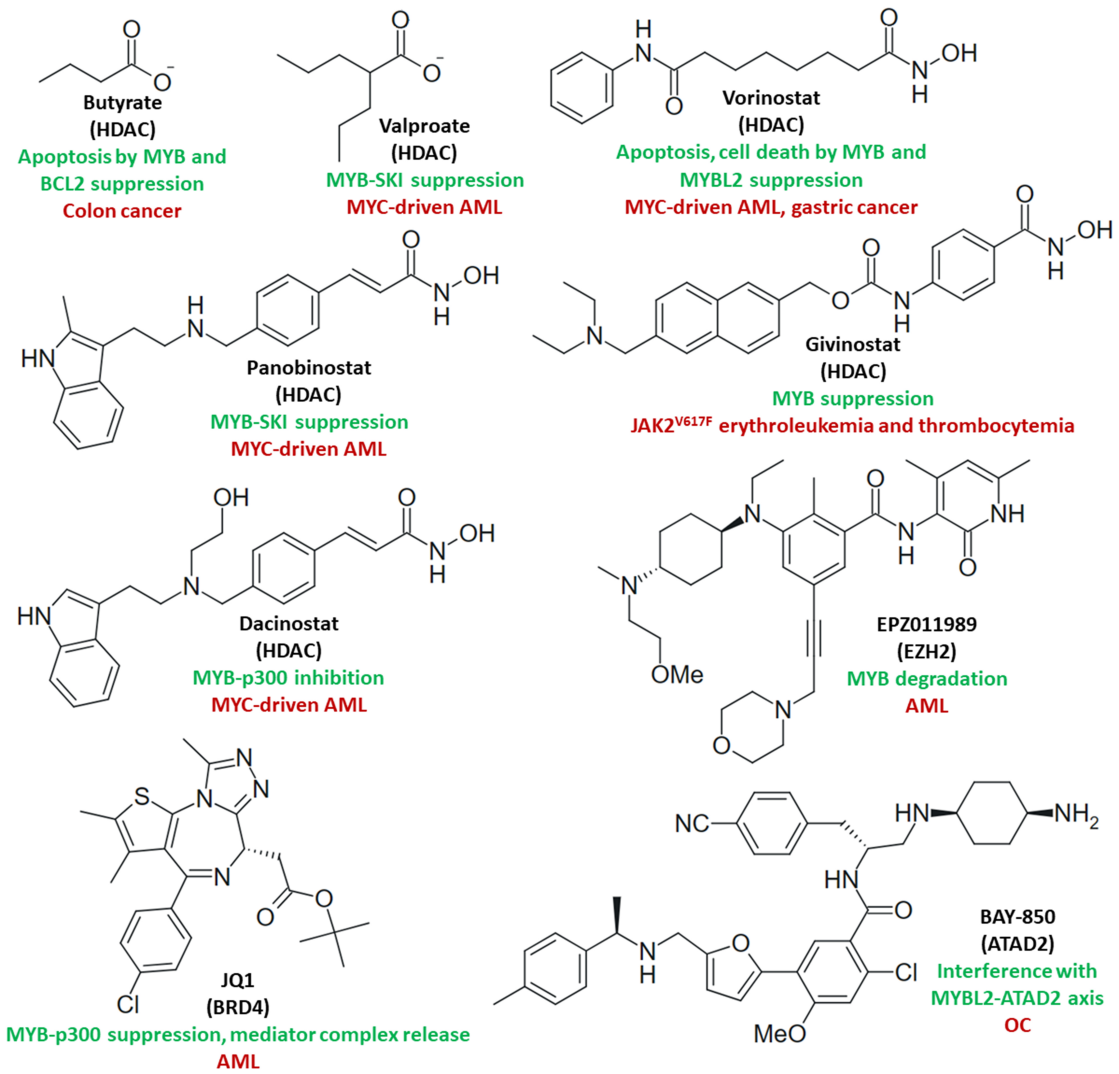
Structures of epigenetic drugs (targets in brackets) with effects on MYB activity and/or MYB-dependent cancers (green: mechanisms; red: targeted cancers).

Various epigenetic drugs such as HDAC inhibitors exhibited suppressive effects on MYB proteins [Figure 6]. Butyrate-mediated differentiation and apoptosis were accompanied by MYB and BCL2 suppression in colon cancers, and BCL2-mediated protection from apoptosis was overcome by butyrate^[192]^. MYB and its target SKI (a transforming growth factor β signaling inhibitor) were responsible for the inhibition of AML differentiation, and the treatment of MYC-driven HL-60 and U937 AML cells with valproic acid or panobinostat suppressed the levels of MYB and SKI^[193]^. Vorinostat induced apoptosis in U937 cells by downregulation of MYB and MYBL2, and cell death upon MYB suppression in gastric cancer cells^[194,195]^. Vorinostat was highly active against MYB-TYK2 B-ALL cells *in vitro* and suppressed MYB-TYK2 B-ALL burden in mice^[175]^. A phase 2 study of vorinostat with advanced, recurrent, or metastatic ACC showed encouraging results with high clinical benefit and 6-month stable disease rates^[196]^. Givinostat-mediated MYB suppression inhibited cell proliferation and induced apoptosis in gain-of-function JAK2^V617F^-mutant HEL erythroleukemia and UKE1 essential thrombocythemia cells^[197]^. The specific JAK2 inhibitor TG101209 is also a MYB suppressor in Burkitt lymphoma, and the JAK1/2 inhibitor ruxolitinib is currently applied for the treatment of JAK-STAT-dependent neoplasms (including JAK2^V617F^ forms), but with limited efficacy based on cytoprotective autophagy induction both in JAK2-wildtype (HL-60) and in JAK2^V617F^ cells (HEL)^[176,198]^. Thus, the lethal effects of givinostat on JAK2^V617F^ mutant leukemia cells might be useful in combination with JAK1/2 inhibitors to augment cell death and to prevent resistance formation. Dacinostat (LAQ824) inhibited MYB activity by interfering with p300 binding and the MYB transactivation domain, and induced cell death in MYC-driven HL-60 cells associated with downregulation of MYB^[199]^. The histone methyltransferase enhancer of zeste homolog 2 (EZH2) is a target of MYB and both factors were downregulated by the EZH2 inhibitor EPZ011989 in AML cells via proteasomal degradation, which was accompanied by apoptosis induction and G2/M arrest. In addition, EZH2 inhibition blocked AML progression *in vivo,* leading to prolonged animal survival^[200]^. The diazepine-based bromodomain and extra-terminal protein (BET) inhibitor JQ1 suppressed functional MYB-p300 in AML cells by inhibition of the reader protein bromodomain-containing protein 4 (BRD4)^[201]^. JQ1-mediated BRD4 inhibition led to mediator complex release from MYB target genes in AML cells^[202]^. Proteolysis-targeting chimeras (PROTACs) were prepared from JQ1 linked with an E3 ubiquitin ligase cereblon (CRBN)-targeting phthalimide. The PROTAC ARV-825 was antiproliferative in a panel of 13 multiple myeloma (MM) cell lines, led to cell cycle arrest and apoptosis, and quickly degraded BRD2 and BRD4 accompanied by suppression of MYB and MYC. ARV-825 also inhibited MM growth and led to prolonged animal survival in MM xenografts^[203]^. Taken together, various epigenetic drugs are able to downregulate MYB in AML and MM in line with their ability to induce apoptosis and cell cycle arrest. The PROTAC dBET6, which differs from ARV-825 in its linker system, decreased BRD4 and MYB levels in patient-derived MYB-NFIB and MYB1-NFIB ACC cell lines (SG28 and SG32) and suppressed ACC growth *in vitro* and *in vivo*^[204]^. ATPase family AAA domain containing 2 (ATAD2) is a chromatin-modifying BRD protein, which was upregulated in OC via MYBL2, and the blocking of the MYBL2-ATAD2 axis by the ATAD2 inhibitor and substituted furan derivative BAY-850 was strongly antiproliferative in A2780 and SKOV3 OC cells, as well as in taxol-resistant SKOV3/TAX cells^[205]^.

#### Modulators of transcription factors and MYB gene expression

Activation of RAR and RXR by RA (vitamin A) suppressed v-MYB transactivation in BM2 cells^[88]^. RA also downregulated MYB in a MYB-transgenic zebrafish ACC model^[206]^. ATRA upregulated NOTCH1 intracellular structural domain 1 (NICD1) and suppressed MYB via activation of RARα followed by inhibition of lung metastasis formation by highly metastatic SACC-LM cells^[207]^. Strong tumor growth inhibition and MYB downregulation were observed for RA in MYB-NFIB and PIK3CA^R88Q^ mutant patient-derived ACC in combination with the PI3K inhibitor alpelisib^[164]^. A phase 2 trial of ATRA in advanced ACC showed a correlation between suppressed MYB and prolonged stable disease as a response to ATRA, but patients with low MYB expression showed better responses (longer PFS) to ATRA therapy than patients with high MYB levels, suggesting a more efficient MYB-suppressing activity of ATRA when MYB expression is low^[208]^. In addition, MYB regulates the nuclear receptor vitamin D receptor (VDR), and 1,25-dihydroxyvitamin D3 (calcitriol) was described as a differentiation inducer in HL-60 and M1 leukemia cells by suppression of MYB^[209,210]^. Vitamin D is administered as a supplement to patients receiving imatinib therapy because imatinib leads to suppression of calcium metabolism and calcitriol production. A clinical study with CML patients receiving imatinib plus calcitriol showed a stabilization of the vitamin D levels, albeit without visible anticancer effects compared with imatinib alone^[211]^. A new preclinical study using the more active calcitriol analog inecalcitol synergistically enhanced the anticancer activities of imatinib and dasatinib against CML cells *in vitro* and might become a more suitable combination partner for these kinase inhibitors in future clinical trials for CML^[212]^.

Resistance to the GR activator dexamethasone was associated with increased MYB and BCL2 expression in ALL^[93]^. However, dexamethasone downregulated MYB and MYC via activation of GR and miR-103 in CEM-C7H2 ALL cells^[96]^. In addition, dexamethasone was able to suppress MYB in myeloid leukemia cells^[209]^. Since MYC upregulation was associated with resistance to GCs, the inhibitory effects of dexamethasone on the MYC activator MYB can contribute to a better understanding of GC resistance and provide hints at resistance circumvention mechanisms. The treatment of COLO-205 CRC cells with the ER agonist estradiol led to apoptosis induction via MYB and BCL2 suppression^[213]^. Notably, the hormone estradiol drives ER-positive breast cancers, but its anticancer effects on CRC upon MYB suppression deserve more investigation. Selective aryl hydrocarbon receptor (AhR ) agonists (e.g., semaxanib) blocked IGF-2-mediated cell growth and reduced MYB expression in MCF-7 breast cancer cells^[214]^. The remarkable effect of AhR agonists on IGF signaling might also be applicable for combination therapies with IGF-1R inhibitors in other MYB-dependent cancers such as ACC.

**Figure 7:**
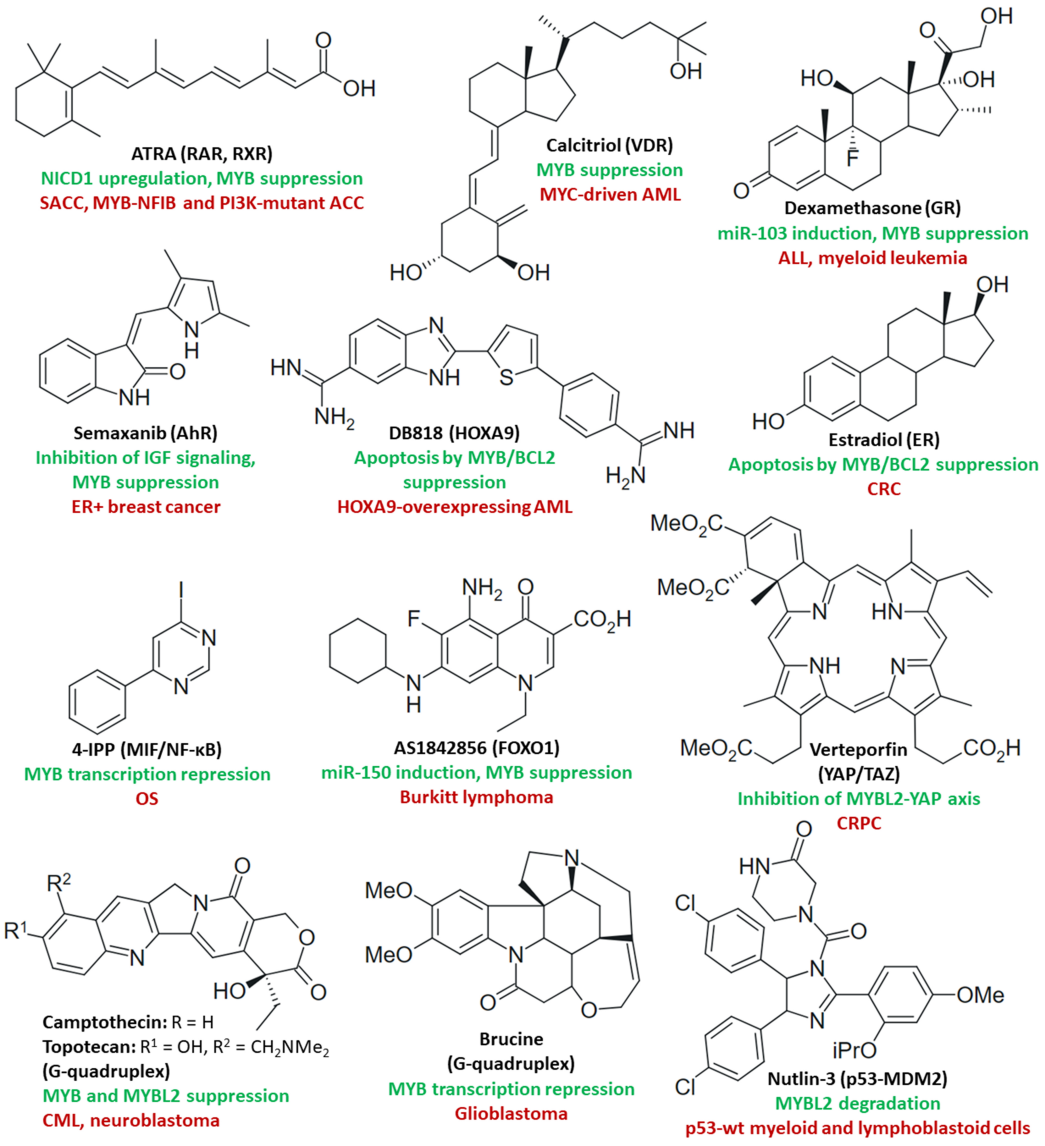
Structures of transcription factor modulators and MYB promoter G-quadruplex binders (targets in brackets) with effects on MYB activity and/or MYB-dependent cancers (green: mechanisms; red: targeted cancers).

In CRPC, the activity of the transcription co-regulator yes-associated protein 1 (YAP1) was induced by MYBL2; however, treatment with the porphyrin-based YAP/TAZ (transcriptional coactivator with PDZ-binding motif) inhibitor verteporfin reversed resistance to androgen depletion therapy and prevented bone metastasis formation *in vivo*^[108]^. Overexpression of the transcription factor homeobox A9 (HOXA9) was associated with poor prognosis for leukemias, including AML^[215]^. DB818 is an inhibitor of the AML driving HOXA9, which led to growth inhibition and apoptosis induction in HOXA9-overexpressing OCI/AML3, MV4-11, and THP-1 AML cells by suppression of MYB, MYC, and BCL2^[216]^. The quinoline-based forkhead box protein O1 (FOXO1) inhibitor AS1842856 blocked cell growth, induced apoptosis, and suppressed MYB by upregulation of the tumor suppressor miR-150 in BL-41 and Namalwa Burkitt lymphoma cells^[217]^. In OS cells, the migration inhibitory factor (MIF) inhibitor 4-iodo-6-phenylpyrimidine (4-IPP) reduced proliferation and metastasis by suppression of MIF/CD74-induced NF-κB/positive transcription elongation factor b (P-TEFb ) complex-mediated MYB transcription^[218]^. The topoisomerase I inhibitory alkaloid camptothecin and its derivative topotecan can bind to the G-quadruplex enhancer of MYB, thus inhibiting the expression of MYB in K562 CML cells^[219,220]^. The alkaloid brucine also binds to G-quadruplex sequences of the MYB promoter, leading to MYB suppression, U87 glioblastoma growth inhibition, and cell cycle arrest^[221]^. The p53 activator Nutlin-3 induced MYBL2 degradation and G1 phase block by inhibition of the p53 suppressor MDM2 in primary leukemia cells as well as in p53-wildtype myeloid (OCI, MOLM) and lymphoblastoid cells (SKW6.4, EHEB) via miR-34a. These effects were not observed upon treatment with the alkylating agent chlorambucil, indicating a superior efficacy of Nutlin-3 against these leukemia cells^[222]^. Figure 7 shows the structures of the described transcription factor modulators and MYB G-quadruplex binders.

#### Natural products and their (semi-)synthetic analogs

**Figure 8:**
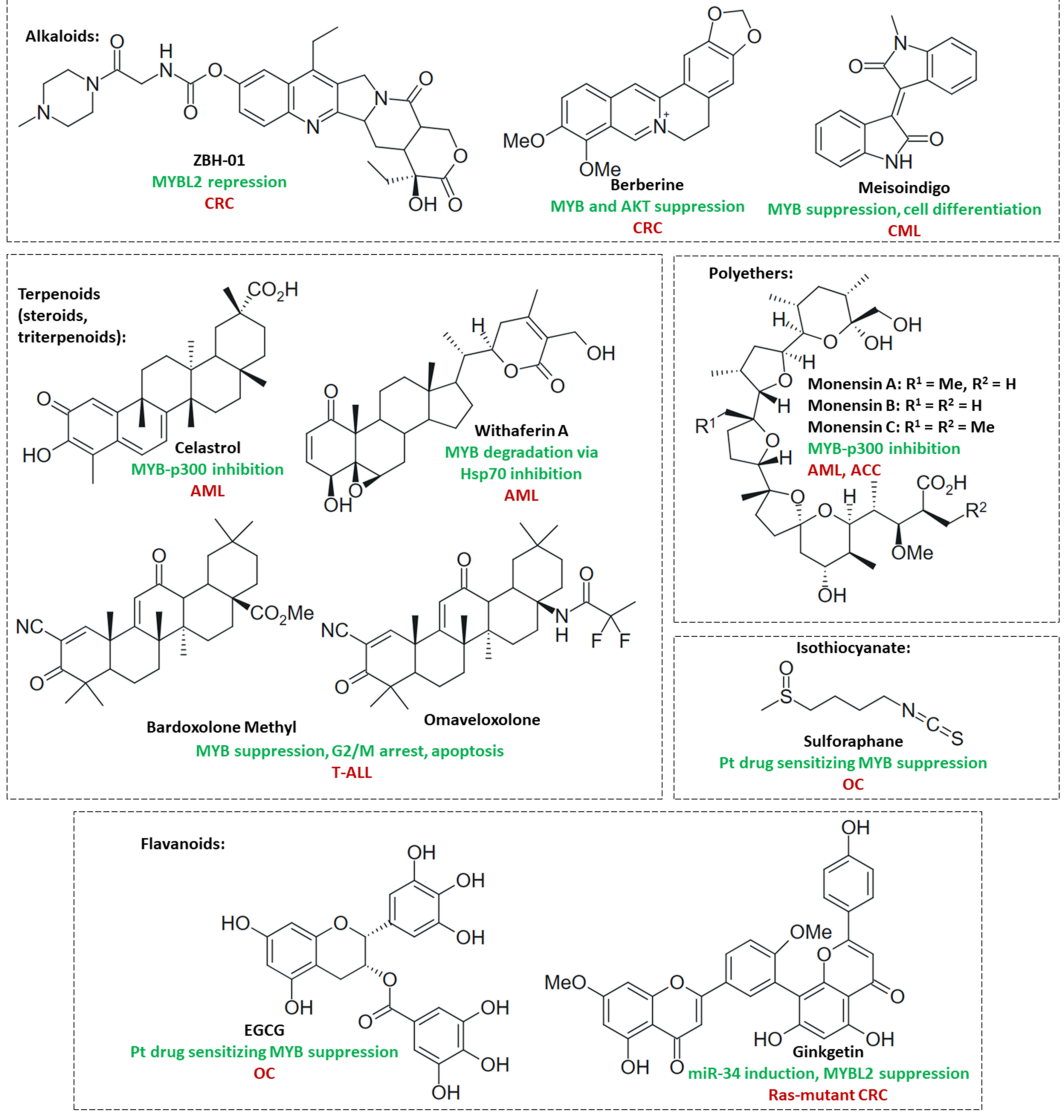
Structures of natural products (arranged according to compound classes) with inhibitory effects on MYB activity (for the terpenoids RA/ATRA, calcitriol, and steroids dexamethasone and estradiol, as well as the alkaloids camptothecin and topotecan see Figure 7; green: mechanisms; red: targeted cancers). RA: Retinoic acid; ATRA: all-*trans* retinoic acid.

Natural products such as retinoids and steroids were shown to affect MYB expression [Figure 7]. The alkaloids camptothecin and topotecan, which reportedly interacted with G-quadruplex regions of the MYB promoter and led to MYB suppression, also suppressed MYBL2 and MYCN expression in IMR-32, Kelly, and LAN-5 neuroblastoma cells accompanied by apoptosis induction^[223]^. Repression of MYBL2 was likewise observed in LS174T and SW1116 CRC cells treated with the camptothecin analog ZBH-01^[224]^. The combination of the alkaloid berberine with oligomeric proanthocyanidines revealed synergistic antiproliferative and pro-apoptotic effects in RKO and HT-29 CRC cells based on MYB and AKT downregulation, thus indicating an important role of MYB in the eminent chemoresistance-associated PI3K-AKT signaling pathway^[225]^. Natural MYB-p300 inhibitors, either by direct interaction with the MYB-p300 complex or by indirect mechanisms, were summarized recently, and naphthoquinones (naphthazarin, plumbagin, and shikonin), sesquiterpenes (helenalin acetate, mexicanin, and warburganal), the triterpene celastrol, and the steroid lactone withaferin A exhibited notable effects^[226,227]^. In particular, the MYB-p300 inhibitor celastrol directly blocked the MYB-KIX (kinase-inducible domain interacting domain) interaction of the MYB-p300 complex and showed antiproliferative activity against AML cells along with prolonged survival in an AML mouse model^[228]^. Withaferin A was active against a panel of AML cell lines (OCI-AML3, MV4-11, SHI1, and U937) upon MYB ablation by interference with Hsp70 followed by induction of UPR and MYB degradation^[229]^. Of note, the Hsp90 inhibitor tanespimycin was active against MYB-TYK2 fusion B-cell ALL cells^[175]^. Thus, protein stability mediated by heat-shock proteins/chaperones appears to be a vital mechanism and a promising drug target in MYB-driven leukemia. T-ALL cells (MOLT-4, CCRF-CEM, P12-ICHIKAWA, and RPMI-8402 cell lines) treated with the synthetic oleanane triterpenes bardoxolone methyl and omaveloxolone showed suppressed MYB expression associated with cell growth inhibition, G2/M arrest, and apoptosis induction. Both oleanane triterpenes also sensitized MOLT-4 cells to doxorubicin^[230]^. MYB suppression by the dietary factors epigallocatechin gallate (EGCG) and sulforaphane sensitized OC cells to cisplatin^[39]^. The antibiotic polyether monensin was described as a MYB inhibitor with antiproliferative activity against AML and ACC cells, but the inhibition of the MYB-p300 interaction by monensin occurred indirectly and not by targeting MYB-KIX^[231]^. The ginkgo biflavanoid ginkgetin caused apoptosis and G2/M arrest in Ras-mutant HCT-116 CRC cells and inhibited HCT-116 xenograft growth. Ginkgetin induced miR-34a, leading to downregulation of MYBL2, CDK1, and cyclin B1, and might be a suitable therapy option for Ras-mutant CRCs^[232]^. The indirubin derivative meisoindigo showed activity against CML and induced differentiation of ML-1 myeloblastic leukemia cells by MYB suppression^[233]^. It is noteworthy that a Ph^+^ CML patient treated with indirubin or meisoindigo had stable disease for 32 years before imatinib therapy for three months led to a complete response^[234]^. Thus, a combination of imatinib with meisoindigo appears to be reasonable for future CML clinical studies. Figure 8 shows the structures of natural products with MYB-inhibitory activity.

Compounds with 3,4,5-trimethoxyphenyl-related motifs appear to be especially promising MYB inhibitors [Figure 8]. The natural 3,4,5-trimethoxycinnamide piperlongumine induced apoptosis in a panel of B-ALL cell lines (including GC-resistant cell lines) by suppression of anti-apoptotic factors such as MYB^[235]^. The podophyllotoxin-derived topoisomerase II inhibitors etoposide and teniposide inhibited MYB activity in a cell-based screening assay and promoted MYB degradation in MYC-driven NB4, HL-60, and U937 AML cells^[236]^. Screening of synthetic 3,4,5-trimethoxyphenyl derivatives identified the tubulin-binding naphthopyran BCR-TMP as a strong inhibitor of MYB activity with high antiproliferative activity against MLL-AF9 transformed AML cells and patient-derived ACC cells. BCR-TMP inhibited MYB transactivation by interference with p300 interaction, albeit probably not by targeting the KIX domain, and promoted MYB degradation^[237]^. Chemical fine-tuning of the BCR-TMP structure led to further halogen-substituted analogs with high antiproliferative and antiangiogenic activities against a panel of solid tumor cell lines (including multidrug-resistant cell lines) by combined MYB- and tubulin-targeting mechanisms^[238,239]^.

**Figure 9:**
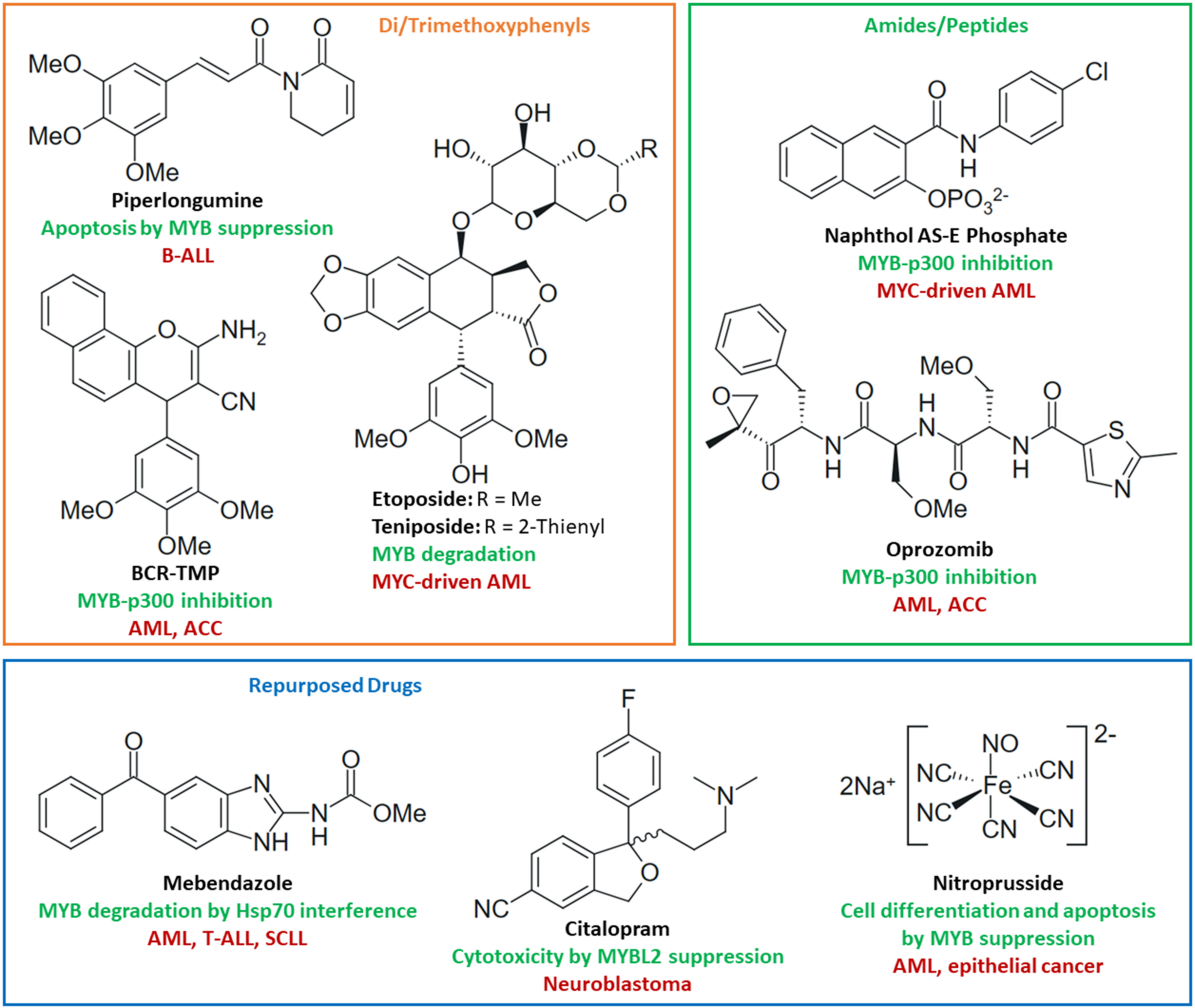
Structures of small-molecule di/trimethoxyphenyl- and amide/peptide-based inhibitors (polypeptides were omitted for clarity) and repurposed drugs with inhibitory effects on MYB activity (green: mechanisms; red: targeted cancers).

Notably, the synthetic naphthalene-based KIX domain inhibitor naphthol AS-E phosphate was the first small-molecule amide compound identified as a direct MYB-p300 inhibitor in MYC-driven AML cell lines [Figure 9]^[240]^. However, MYB-KIX-targeting peptides were also developed. The peptidomimetic MYBMIM peptide was designed to mimic the native MYB residues 293-310, which play a crucial role in the interaction of MYB with the p300 KIX domain. MYBMIM led to MYB-p300 dissociation, suppression of MYB activity followed by downregulation of MYC and BCL2, apoptosis induction in AML cells, and prolonged survival in patient-derived MLL-rearranged leukemia mouse model^[241]^. The dual CBP/p300 KIX dual site inhibitor MybLL-tide exhibited picomolar activity and suppressed MYB genes in AML^[242]^. The synthetic peptide-based proteasome inhibitor oprozomib also prevented p300-mediated activation of MYB, leading to antiproliferative activity against AML and ACC cells. However, the inhibition of MYB-p300 by oprozomib was probably indirect and not by MYB-KIX targeting [Figure 9]^[243]^.

#### Repurposed drugs

The repurposing of known drugs as MYB inhibitors is a promising strategy [Figure 9]. The anthelmintic benzimidazole derivative mebendazol suppressed AML cell colony formation and progression in mice based on interference with Hsp70, which promoted proteasomal MYB degradation^[244]^. As mentioned above, this effect was also observed for the natural MYB inhibitor withaferin A. In addition, mebendazole was active (*in vitro* and *in vivo*) against MYB-regulated T-cell acute lymphocytic leukemia protein 1 (TAL1) 5’super-enhancer (5’SE) mutant T-ALL Jurkat and patient-derived cells by enhanced MYB degradation^[245]^. In BBC2 and KG1 stem cell leukemia and lymphoma cells, the tumor suppressor miR-150-5p was downregulated by MYB and other factors [fibroblast growth factor receptor 1 (FGFR1) and MYC], but treatment with mebendazole showed increased antiproliferative activity and apoptosis induction. Mebendazole also led to prolonged survival in mice with FGFR1-dependent leukemia^[246]^. Suppression of miR-150-5p was correlated with imatinib resistance in CML, which can be overcome by mebendazole^[130,247]^. The antidepressant citalopram exhibited cytotoxic activity and strongly suppressed MYBL2 in neuroblastoma cells (B104, SH-SY5Y, and Kelly)^[248]^. Since MYBL2 expression in neuroblastoma is associated with poor prognosis, citalopram has the potential to become a drug candidate for patients with MYBL2-driven neuroblastoma. Psychiatric medications are often prescribed for cancer patients to cope with anxiety and depression upon diagnosis. Thus, antidepressants can be a valuable source of anticancer drugs^[249]^. The inorganic anti-hypertensive drug sodium nitroprusside is a nitric oxide (NO) donor. NO released by nitroprusside induced differentiation in MYC-dependent HL-60 AML cells and apoptosis in NA epithelial cancer cells by suppression of MYB and MYC^[250,251]^. The downregulation of MYB by the free radical gas NO presents an uncommon mechanism of MYB inhibition, which deserves thorough investigation, including other NO donors.

## CONCLUSION

MYB proteins play a crucial and versatile role in cancer drug resistance. A detailed understanding of the effects of MYB proteins on drug resistance enables a proper adjustment of established cancer therapies and the design of new and more efficient therapies. Of note, cancer treatment with certain drugs such as HDAC and kinase inhibitors, ATRA, and etoposide, which modulate MYB protein expression or activity as part of their anticancer activity, also revealed MYB-mediated resistance mechanisms. This indicates a complex and fine-tuned regulatory network for MYB proteins that contributes to drug sensitivity. The consideration of MYB-associated mechanisms for anticancer drug development will likely improve the therapy options for cancer patients in the future. In addition, MYB-targeting efforts have already revealed several promising anticancer drug candidates for the treatment of various cancers, including strongly MYB-dependent AML and ACC. Further cancers sensitive to MYB-targeted therapy include various leukemias (e.g., BCR/ABL-positive CML and ALL) and lymphomas (DLBCL, Burkitt lymphoma), gynecological cancers (ER-positive breast cancer, OC), gastrointestinal cancers, and CRPC. Aside from MYB and its fusion proteins, MYBL2 was also identified as a drug target. Sensitizing effects and synergies were documented for combinations of MYB-targeting drugs with currently applied cancer therapeutics such as cisplatin, doxorubicin, and imatinib, which underline the potential of MYB inhibitors as promising anticancer drug candidates. However, adverse effects such as cisplatin-mediated ototoxicity can be augmented by MYB suppression, which should be kept in mind when designing clinical studies using combination therapies with MYB inhibitors.

## DECLARATIONS

### Copyright

© The Author(s) 2024.

## References

[1] Gottesman MM, Robey RW, Ambudkar SV (2023). New mechanisms of multidrug resistance: an introduction to the cancer drug resistance special collection. Cancer Drug Resist.

[2] Bushweller JH (2019). Targeting transcription factors in cancer - from undruggable to reality. Nat Rev Cancer.

[3] Vervoort SJ, Devlin JR, Kwiatkowski N, Teng M, Gray NS, Johnstone RW (2022). Targeting transcription cycles in cancer. Nat Rev Cancer.

[4] Chen B, Dragomir MP, Yang C, Li Q, Horst D, Calin GA (2022). Targeting non-coding RNAs to overcome cancer therapy resistance. Signal Transduct Target Ther.

[5] Planey SL, Kumar R, Arnott JA (2013). Post-translational modification of transcription factors: mechanisms and potential therapeutic interventions. Curr Mol Pharmacol.

[6] Vishnoi K, Viswakarma N, Rana A, Rana B (2020). Transcription factors in cancer development and therapy. Cancers.

[7] Martínez-Martín S, Soucek L (2021). MYC inhibitors in multiple myeloma. Cancer Drug Resist.

[8] Martínez-Martín S, Beaulieu ME, Soucek L (2023). Targeting MYC-driven lymphoma: lessons learned and future directions. Cancer Drug Resist.

[9] Singleton KR, Crawford L, Tsui E (2017). Melanoma therapeutic strategies that select against resistance by exploiting MYC-driven evolutionary convergence. Cell Rep.

[10] Cicirò Y, Sala A (2021). MYB oncoproteins: emerging players and potential therapeutic targets in human cancer. Oncogenesis.

[11] Anand S, Vikramdeo KS, Sudan SK (2024). From modulation of cellular plasticity to potentiation of therapeutic resistance: new and emerging roles of MYB transcription factors in human malignancies. Cancer Metastasis Rev.

[12] Nakagoshi H, Kanei-Ishii C, Sawazaki T, Mizuguchi G, Ishii S (1992). Transcriptional activation of the c-myc gene by the c-myb and B-myb gene products. Oncogene.

[13] Duesberg PH, Bister K, Moscovici C (1980). Genetic structure of avian myeloblastosis virus, released from transformed myoblasts as a defective virus particle. Proc Natl Acad Sci USA.

[14] Klempnauer KH, Ramsay G, Bishop JM (1983). The product of the retroviral transforming gene v-myb is a truncated version of the protein encoded by the cellular oncogene c-myb. Cell.

[15] Davidson CJ, Guthrie EE, Lipsick JS (2013). Duplication and maintenance of the Myb genes of vertebrate animals. Biol Open.

[16] Lipsick JS, Manak J, Mitiku N, Chen CK, Fogarty P, Guthrie E (2001). Functional evolution of the Myb oncogene family. Blood Cells Mol Dis.

[17] Ogata K, Hojo H, Aimoto S (1992). Solution structure of a DNA-binding unit of Myb: a helix-turn-helix-related motif with conserved tryptophans forming a hydrophobic core. Proc Natl Acad Sci U S A.

[18] Biedenkapp H, Borgmeyer U, Sippel AE, Klempnauer KH (1988). Viral myb oncogene encodes a sequence-specific DNA-binding activity. Nature.

[19] Quintana AM, Liu F, O’Rourke JP, Ness SA (2011). Identification and regulation of c-Myb target genes in MCF-7 cells. BMC Cancer.

[20] George OL, Ness SA (2014). Situational awareness: regulation of the myb transcription factor in differentiation, the cell cycle and oncogenesis. Cancers.

[21] Ness SA (1999). Myb binding proteins: regulators and cohorts in transformation. Oncogene.

[22] Jin Y, Qi G, Chen G, Wang C, Fan X (2021). Association between B-*Myb* proto-oncogene and the development of malignant tumors. Oncol Lett.

[23] Anand S, Khan MA, Zubair H (2023). MYB sustains hypoxic survival of pancreatic cancer cells by facilitating metabolic reprogramming. EMBO Rep.

[24] Okumura F, Joo-Okumura A, Nakatsukasa K, Kamura T (2017). Hypoxia-inducible factor-2α stabilizes the von Hippel-Lindau (VHL) disease suppressor, Myb-related protein 2. PLoS One.

[25] Zhang Q, Yang H (2012). The roles of VHL-dependent ubiquitination in signaling and cancer. Front Oncol.

[26] Felipe-Abrio B, Verdugo-Sivianes EM, Carnero A (2019). c-MYB- and PGC1a-dependent metabolic switch induced by MYBBP1A loss in renal cancer. Mol Oncol.

[27] Felipe-Abrio B, Carnero A (2020). The tumor suppressor roles of MYBBP1A, a major contributor to metabolism plasticity and stemness. Cancers.

[28] Liu M, Du Q, Mao G, Dai N, Zhang F (2022). MYB proto-oncogene like 2 promotes hepatocellular carcinoma growth and glycolysis via binding to the *Optic atrophy* 3 promoter and activating its expression. Bioengineered.

[29] Yamauchi T, Ishidao T, Nomura T (2008). A B-Myb complex containing clathrin and filamin is required for mitotic spindle function. EMBO J.

[30] Sahara S, Herzog AE, Nör JE (2021). Systemic therapies for salivary gland adenoid cystic carcinoma. Am J Cancer Res.

[31] DeVita VT Jr, Chu E (2008). A history of cancer chemotherapy. Cancer Res.

[32] Sharma I, Sharma A, Tomer R, Negi N, Sobti RC

[33] Vasan N, Baselga J, Hyman DM (2019). A view on drug resistance in cancer. Nature.

[34] Dilruba S, Kalayda GV (2016). Platinum-based drugs: past, present and future. Cancer Chemother Pharmacol.

[35] Rescigno P, Ottaviano M, Palmieri G (2020). Platinum drug sensitivity and resistance in testicular germ cell tumors: two sides of the same coin. Cancer Drug Resist.

[36] Perego P (2021). Tackling cisplatin resistance in ovarian cancer: what can we do?. Cancer Drug Resist.

[37] Funato T, Satou J, Kozawa K, Fujimaki S, Miura T, Kaku M (2001). Use of c-myb antisense oligonucleotides to increase the sensitivity of human colon cancer cells to cisplatin. Oncol Rep.

[38] Miree O, Srivastava SK, Khan MA (2021). Clinicopathologic significance and race-specific prognostic association of MYB overexpression in ovarian cancer. Sci Rep.

[39] Tian M, Tian D, Qiao X, Li J, Zhang L (2019). Modulation of Myb-induced NF-kB -STAT3 signaling and resulting cisplatin resistance in ovarian cancer by dietary factors. J Cell Physiol.

[40] Xue Y, Wu T, Sheng Y, Zhong Y, Hu B, Bao C (2021). MicroRNA-1252-5p, regulated by Myb, inhibits invasion and epithelial-mesenchymal transition of pancreatic cancer cells by targeting NEDD9. Aging.

[41] Wang Y, Zhang CY, Xia RH (2018). The MYB/miR-130a/NDRG2 axis modulates tumor proliferation and metastatic potential in salivary adenoid cystic carcinoma. Cell Death Dis.

[42] Liu W, Fan L, Shao B, Zhang Y (2023). STAT3 promotes migration and invasion of cholangiocarcinoma arising from choledochal cyst by transcriptionally inhibiting miR200c through the c-myb/MEK/ERK signaling pathway. Cell Mol Biol.

[43] Dúcka M, Kučeríková M, Trčka F (2021). c-Myb interferes with inflammatory IL1α-NF-κB pathway in breast cancer cells. Neoplasia.

[44] Zhang XY, Li YF, Ma H, Gao YH (2020). Regulation of MYB mediated cisplatin resistance of ovarian cancer cells involves miR-21-wnt signaling axis. Sci Rep.

[45] Zhou X, Liu M, Deng G (2021). lncRNA LOC102724169 plus cisplatin exhibit the synergistic anti-tumor effect in ovarian cancer with chronic stress. Mol Ther Nucleic Acids.

[46] Bu C, Xu L, Han Y (2022). c-Myb protects cochlear hair cells from cisplatin-induced damage via the PI3K/Akt signaling pathway. Cell Death Discov.

[47] Warner KA, Oklejas AE, Pearson AT (2018). UM-HACC-2A: MYB-NFIB fusion-positive human adenoid cystic carcinoma cell line. Oral Oncol.

[48] Long J, Zhu B, Tian T (2023). Activation of UBEC2 by transcription factor MYBL2 affects DNA damage and promotes gastric cancer progression and cisplatin resistance. Open Med.

[49] Guan X, Meng X, Zhu K (2022). MYSM1 induces apoptosis and sensitizes TNBC cells to cisplatin via RSK3-phospho-BAD pathway. Cell Death Discov.

[50] Niklaus NJ, Humbert M, Tschan MP (2018). Cisplatin sensitivity in breast cancer cells is associated with particular DMTF1 splice variant expression. Biochem Biophys Res Commun.

[51] MacDonald J, Ramos-Valdes Y, Perampalam P, Litovchick L, DiMattia GE, Dick FA (2017). A systematic analysis of negative growth control implicates the DREAM complex in cancer cell dormancy. Mol Cancer Res.

[52] Sampurno S, Bijenhof A, Cheasley D (2013). The Myb-p300-CREB axis modulates intestine homeostasis, radiosensitivity and tumorigenesis. Cell Death Dis.

[53] Liu F, Wang Y, Cao Y (2023). Transcription factor B-MYB activates lncRNA CCAT1 and upregulates SOCS3 to promote chemoresistance in colorectal cancer. Chem Biol Interact.

[54] Kadioglu O, Saeed M, Mahmoud N (2021). Identification of potential novel drug resistance mechanisms by genomic and transcriptomic profiling of colon cancer cells with p53 deletion. Arch Toxicol.

[55] Pekarčíková L, Knopfová L, Beneš P, Šmarda J (2016). c-Myb regulates NOX1/p38 to control survival of colorectal carcinoma cells. Cell Signal.

[56] Mattioli R, Ilari A, Colotti B, Mosca L, Fazi F, Colotti G (2023). Doxorubicin and other anthracyclines in cancers: activity, chemoresistance and its overcoming. Mol Aspects Med.

[57] Melani C, Rivoltini L, Parmiani G, Calabretta B, Colombo MP (1991). Inhibition of proliferation by c-myb antisense oligodeoxynucleotides in colon adenocarcinoma cell lines that express c-myb. Cancer Res.

[58] Sarvaiya PJ, Schwartz JR, Hernandez CP, Rodriguez PC, Vedeckis WV (2012). Role of c-Myb in the survival of pre B-cell acute lymphoblastic leukemia and leukemogenesis. Am J Hematol.

[59] Elcheva IA, Wood T, Chiarolanzio K (2020). RNA-binding protein IGF2BP1 maintains leukemia stem cell properties by regulating HOXB4, MYB, and ALDH1A1. Leukemia.

[60] Říhová K, Dúcka M, Zambo IS (2022). Transcription factor c-Myb: novel prognostic factor in osteosarcoma. Clin Exp Metastasis.

[61] Thorner AR, Hoadley KA, Parker JS, Winkel S, Millikan RC, Perou CM (2009). *In vitro* and *in vivo* analysis of B-Myb in basal-like breast cancer. Oncogene.

[62] Grassilli E, Salomoni P, Perrotti D, Franceschi C, Calabretta B (1999). Resistance to apoptosis in CTLL-2 cells overexpressing B-Myb is associated with B-Myb-dependent bcl-2 induction. Cancer Res.

[63] Inoue K, Fry EA (2018). Tumor suppression by the EGR1, DMP1, ARF, p53, and PTEN Network. Cancer Invest.

[64] Taneja P, Mallakin A, Matise LA, Frazier DP, Choudhary M, Inoue K (2007). Repression of Dmp1 and Arf transcription by anthracyclins: critical roles of the NF-kappaB subunit p65. Oncogene.

[65] Montecucco A, Zanetta F, Biamonti G (2015). Molecular mechanisms of etoposide. EXCLI J.

[66] Lotz C, Lamour V (2020). The interplay between DNA topoisomerase 2α post-translational modifications and drug resistance. Cancer Drug Resist.

[67] Tanno B, Sesti F, Cesi V (2010). Expression of Slug is regulated by c-Myb and is required for invasion and bone marrow homing of cancer cells of different origin. J Biol Chem.

[68] Levenson VV, Davidovich IA, Roninson IB (2000). Pleiotropic resistance to DNA-interactive drugs is associated with increased expression of genes involved in DNA replication, repair, and stress response. Cancer Res.

[69] Singh N, Miner A, Hennis L, Mittal S (2021). Mechanisms of temozolomide resistance in glioblastoma - a comprehensive review. Cancer Drug Resist.

[70] Siebzehnrubl FA, Silver DJ, Tugertimur B (2013). The ZEB1 pathway links glioblastoma initiation, invasion and chemoresistance. EMBO Mol Med.

[71] Pieraccioli M, Imbastari F, Antonov A, Melino G, Raschellà G (2013). Activation of miR200 by c-Myb depends on ZEB1 expression and miR200 promoter methylation. Cell Cycle.

[72] Hugo HJ, Pereira L, Suryadinata R (2013). Direct repression of MYB by ZEB1 suppresses proliferation and epithelial gene expression during epithelial-to-mesenchymal transition of breast cancer cells. Breast Cancer Res.

[73] Bhise NS, Chauhan L, Shin M (2015). MicroRNA-mRNA pairs associated with outcome in AML: from in vitro cell-based studies to AML patients. Front Pharmacol.

[74] Paik J (2021). Olaparib: A review as first-line maintenance therapy in advanced ovarian cancer. Target Oncol.

[75] Qi G, Zhang C, Ma H (2021). CDCA8, targeted by MYBL2, promotes malignant progression and olaparib insensitivity in ovarian cancer. Am J Cancer Res.

[76] Li L, Chang W, Yang G (2014). Targeting poly(ADP-ribose) polymerase and the c-Myb-regulated DNA damage response pathway in castration-resistant prostate cancer. Sci Signal.

[77] Murray V, Chen JK, Chung LH (2018). The interaction of the metallo-glycopeptide anti-tumour drug bleomycin with DNA. Int J Mol Sci.

[78] Robertson KA, Bullock HA, Xu Y (2001). Altered expression of Ape1/ref-1 in germ cell tumors and overexpression in NT2 cells confers resistance to bleomycin and radiation. Cancer Res.

[79] Kim TE, Chang JE (2023). Recent studies in photodynamic therapy for cancer treatment: from basic research to clinical trials. Pharmaceutics.

[80] Hui YJ, Chen H, Peng XC (2023). Up-regulation of ABCG2 by MYBL2 deletion drives chlorin e6-mediated photodynamic therapy resistance in colorectal cancer. Photodiagnosis Photodyn Ther.

[81] Mo W, Zhang JT (2012). Human ABCG2: structure, function, and its role in multidrug resistance. Int J Biochem Mol Biol.

[82] Škubník J, Pavlíčková VS, Ruml T, Rimpelová S (2021). Vincristine in combination therapy of cancer: emerging trends in clinics. Biology.

[83] Chen L, Xu X, Wang J (2005). [Spectrum of gene expression of a multi-drug resistant leukemia cell line with high tumorigenecity in nude mice]. Zhonghua Zhong Liu Za Zhi.

[84] Mosca L, Ilari A, Fazi F, Assaraf YG, Colotti G (2021). Taxanes in cancer treatment: activity, chemoresistance and its overcoming. Drug Resist Updat.

[85] Chen L, Song Y, Hou T (2022). Circ_0004087 interaction with SND1 promotes docetaxel resistance in prostate cancer by boosting the mitosis error correction mechanism. J Exp Clin Cancer Res.

[86] Wei Y, Yang C, Wei J, Li W, Qin Y, Liu G (2023). Identification and verification of microtubule associated genes in lung adenocarcinoma. Sci Rep.

[87] Hunsu VO, Facey COB, Fields JZ, Boman BM (2021). Retinoids as chemo-preventive and molecular-targeted anti-cancer therapies. Int J Mol Sci.

[88] Smarda J, Zemanová K, Bryja J (1999). Retinoid X receptor suppresses transformation by the v-myb oncogene. J Leukoc Biol.

[89] Benes P, Macecková V, Zatloukalová J, Kovárová L, Smardová J, Smarda J (2007). Retinoic acid enhances differentiation of v-myb-transformed monoblasts induced by okadaic acid. Leuk Res.

[90] Trčka F, Šmarda J, Knopfová L, Kuziaková K, Beneš P (2013). Nuclear factor of activated T-cells 1 increases sensitivity of v-myb transformed monoblasts to all-trans retinoic acid. Cell Signal.

[91] Cesi V, Tanno B, Vitali R (2002). Cyclin D1-dependent regulation of B-myb activity in early stages of neuroblastoma differentiation. Cell Death Differ.

[92] Olivas-Aguirre M, Torres-López L, Pottosin I, Dobrovinskaya O (2021). Overcoming glucocorticoid resistance in acute lymphoblastic leukemia: repurposed drugs can improve the protocol. Front Oncol.

[93] Jing D, Bhadri VA, Beck D (2015). Opposing regulation of BIM and BCL2 controls glucocorticoid-induced apoptosis of pediatric acute lymphoblastic leukemia cells. Blood.

[94] Salomoni P, Perrotti D, Martinez R, Franceschi C, Calabretta B (1997). Resistance to apoptosis in CTLL-2 cells constitutively expressing c-Myb is associated with induction of BCL-2 expression and Myb-dependent regulation of the bcl-2 promoter activity. Proc Natl Acad Sci USA.

[95] Geng CD, Vedeckis WV (2010). Use of recombinant cell-permeable small peptides to modulate glucocorticoid sensitivity of acute lymphoblastic leukemia cells. Biochemistry.

[96] Kfir-Erenfeld S, Haggiag N, Biton M, Stepensky P, Assayag-Asherie N, Yefenof E (2017). miR-103 inhibits proliferation and sensitizes hemopoietic tumor cells for glucocorticoid-induced apoptosis. Oncotarget.

[97] Drabsch Y, Hugo H, Zhang R (2007). Mechanism of and requirement for estrogen-regulated *MYB* expression in estrogen-receptor-positive breast cancer cells. Proc Natl Acad Sci U S A.

[98] Howell A, Howell SJ (2023). Tamoxifen evolution. Br J Cancer.

[99] Gao Y, Zhang W, Liu C, Li G (2019). miR-200 affects tamoxifen resistance in breast cancer cells through regulation of MYB. Sci Rep.

[100] Hodges LC, Cook JD, Lobenhofer EK (2003). Tamoxifen functions as a molecular agonist inducing cell cycle-associated genes in breast cancer cells. Mol Cancer Res.

[101] Li X, Zhang X, Wu CC (2021). The role of MYB proto-oncogene like 2 in tamoxifen resistance in breast cancer. J Mol Histol.

[102] Nathan MR, Schmid P (2017). A review of fulvestrant in breast cancer. Oncol Ther.

[103] Lee J, Hirsh AS, Wittner BS (2011). Induction of stable drug resistance in human breast cancer cells using a combinatorial zinc finger transcription factor library. PLoS One.

[104] Edavana VK, Penney RB, Yao-Borengasser A (2013). Fulvestrant up regulates *UGT1A4* and *MRPs* through ERα and c-Myb pathways: a possible primary drug disposition mechanism. Springerplus.

[105] Edwards J, Krishna NS, Witton CJ, Bartlett JMS (2003). Gene amplifications associated with the development of hormone-resistant prostate cancer. Clin Cancer Res.

[106] Thompson TC, Li L (2014). New targets for resistant prostate cancer. Oncotarget.

[107] Srivastava SK, Bhardwaj A, Singh S (2012). Myb overexpression overrides androgen depletion-induced cell cycle arrest and apoptosis in prostate cancer cells, and confers aggressive malignant traits: potential role in castration resistance. Carcinogenesis.

[108] Li Q, Wang M, Hu Y (2021). MYBL2 disrupts the Hippo-YAP pathway and confers castration resistance and metastatic potential in prostate cancer. Theranostics.

[109] Srivastava SK, Khan MA, Anand S (2022). MYB interacts with androgen receptor, sustains its ligand-independent activation and promotes castration resistance in prostate cancer. Br J Cancer.

[110] (2011). de Bono JS, Logothetis CJ, Molina A, et al; COU-AA-301 Investigators. Abiraterone and increased survival in metastatic prostate cancer. N Engl J Med.

[111] Blatti C, de la Fuente J, Gao H (2023). Bayesian machine learning enables identification of transcriptional network disruptions associated with drug-resistant prostate cancer. Cancer Res.

[112] Baudino TA (2015). Targeted cancer therapy: the next generation of cancer treatment. Curr Drug Discov Technol.

[113] Nepali K, Liou JP (2021). Recent developments in epigenetic cancer therapeutics: clinical advancement and emerging trends. J Biomed Sci.

[114] Waldman AD, Fritz JM, Lenardo MJ (2020). A guide to cancer immunotherapy: from T cell basic science to clinical practice. Nat Rev Immunol.

[115] Steins M, Thomas M, Geißler M

[116] García-Foncillas J, Sunakawa Y, Aderka D (2019). Distinguishing features of cetuximab and panitumumab in colorectal cancer and other solid tumors. Front Oncol.

[117] Liang L, Liu M, Sun X (2021). Identification of key genes involved in tumor immune cell infiltration and cetuximab resistance in colorectal cancer. Cancer Cell Int.

[118] Iida M, Brand TM, Campbell DA, Li C, Wheeler DL (2013). Yes and Lyn play a role in nuclear translocation of the epidermal growth factor receptor. Oncogene.

[119] Garnock-Jones KP, Keating GM, Scott LJ (2010). Trastuzumab: a review of its use as adjuvant treatment in human epidermal growth factor receptor 2 (HER2)-positive early breast cancer. Drugs.

[120] Rezaei Z, Dastjerdi K, Allahyari A (2023). Plasma microRNA-195, -34c, and -1246 as novel biomarkers for the diagnosis of trastuzumab-resistant HER2-positive breast cancer patients. Toxicol Appl Pharmacol.

[121] Iyer R, Fetterly G, Lugade A, Thanavala Y (2010). Sorafenib: a clinical and pharmacologic review. Expert Opin Pharmacother.

[122] Zhu J, Wu Y, Yu Y, Li Y, Shen J, Zhang R (2022). MYBL1 induces transcriptional activation of ANGPT2 to promote tumor angiogenesis and confer sorafenib resistance in human hepatocellular carcinoma. Cell Death Dis.

[123] Okumura F, Uematsu K, Byrne SD (2016). Parallel regulation of von hippel-lindau disease by pVHL-mediated degradation of B-Myb and hypoxia-inducible factor α. Mol Cell Biol.

[124] Kanei-Ishii C, Nomura T, Takagi T, Watanabe N, Nakayama KI, Ishii S (2008). Fbxw7 acts as an E3 ubiquitin ligase that targets c-Myb for nemo-like kinase (NLK)-induced degradation. J Biol Chem.

[125] Iqbal N, Iqbal N (2014). Imatinib: a breakthrough of targeted therapy in cancer. Chemother Res Pract.

[126] Ohmine K, Nagai T, Tarumoto T (2003). Analysis of gene expression profiles in an imatinib-resistant cell line, KCL22/SR. Stem Cells.

[127] Corradini F, Cesi V, Bartella V (2005). Enhanced proliferative potential of hematopoietic cells expressing degradation-resistant c-Myb mutants. J Biol Chem.

[128] Corradini F, Bussolari R, Cerioli D, Lidonnici MR, Calabretta B (2007). A degradation-resistant c-Myb mutant cooperates with Bcl-2 in enhancing proliferative potential and survival of hematopoietic cells. Blood Cells Mol Dis.

[129] Cofre J, Menezes JR, Pizzatti L, Abdelhay E (2012). Knock-down of Kaiso induces proliferation and blocks granulocytic differentiation in blast crisis of chronic myeloid leukemia. Cancer Cell Int.

[130] Srutova K, Curik N, Burda P (2018). BCR-ABL1 mediated miR-150 downregulation through MYC contributed to myeloid differentiation block and drug resistance in chronic myeloid leukemia. Haematologica.

[131] Fehr A, Arvidsson G, Nordlund J, Lönnerholm G, Stenman G, Andersson MK (2023). Increased MYB alternative promoter usage is associated with relapse in acute lymphoblastic leukemia. Genes Chromosomes Cancer.

[132] Horwitz SM, Feldman TA, Hess BT (2018). The novel SYK/JAK inhibitor cerdulatinib demonstrates good tolerability and clinical response in a phase 2a study in relapsed/refractory peripheral T-cell lymphoma and cutaneous T-cell lymphoma. Blood.

[133] (2021). Tavakoli Shirazi P, Eadie LN, Page EC, Heatley SL, Bruning JB, White DL. Constitutive JAK/STAT signaling is the primary mechanism of resistance to JAKi in TYK2-rearranged acute lymphoblastic leukemia. Cancer Lett.

[134] Angius G, Tomao S, Stati V, Vici P, Bianco V, Tomao F (2020). Prexasertib, a checkpoint kinase inhibitor: from preclinical data to clinical development. Cancer Chemother Pharmacol.

[135] Branigan TB, Kozono D, Schade AE (2021). MMB-FOXM1-driven premature mitosis is required for CHK1 inhibitor sensitivity. Cell Rep.

[136] Chambers AE, Banerjee S, Chaplin T (2003). Histone acetylation-mediated regulation of genes in leukaemic cells. Eur J Cancer.

[137] Drabsch Y, Robert RG, Gonda TJ (2010). MYB suppresses differentiation and apoptosis of human breast cancer cells. Breast Cancer Res.

[138] Marks PA (2007). Discovery and development of SAHA as an anticancer agent. Oncogene.

[139] Peart MJ, Smyth GK, van Laar RK (2005). Identification and functional significance of genes regulated by structurally different histone deacetylase inhibitors. Proc Natl Acad Sci U S A.

[140] Ye P, Zhao L, McGirr C, Gonda TJ (2014). MYB down-regulation enhances sensitivity of U937 myeloid leukemia cells to the histone deacetylase inhibitor LBH589 in vitro and in vivo. Cancer Lett.

[141] Esfahani K, Roudaia L, Buhlaiga N, Del Rincon SV, Papneja N, Miller WH Jr (2020). A review of cancer immunotherapy: from the past, to the present, to the future. Curr Oncol.

[142] Millen R, Malaterre J, Cross RS (2016). Immunomodulation by MYB is associated with tumor relapse in patients with early stage colorectal cancer. Oncoimmunology.

[143] Pan B, Wan T, Zhou Y (2023). The MYBL2-CCL2 axis promotes tumor progression and resistance to anti-PD-1 therapy in ovarian cancer by inducing immunosuppressive macrophages. Cancer Cell Int.

[144] Citro G, Perrotti D, Cucco C (1992). Inhibition of leukemia cell proliferation by receptor-mediated uptake of c-myb antisense oligodeoxynucleotides. Proc Natl Acad Sci U S A.

[145] Citro G, Szczylik C, Ginobbi P, Zupi G, Calabretta B (1994). Inhibition of leukaemia cell proliferation by folic acid-polylysine-mediated introduction of c-myb antisense oligodeoxynucleotides into HL-60 cells. Br J Cancer.

[146] Del Bufalo D, Cucco C, Leonetti C (1996). Effect of cisplatin and c-myb antisense phosphorothioate oligodeoxynucleotides combination on a human colon carcinoma cell line in vitro and in vivo. Br J Cancer.

[147] Hu D, Shao W, Liu L (2021). Intricate crosstalk between MYB and noncoding RNAs in cancer. Cancer Cell Int.

[148] Pham T, Carpinteri S, Sampurno S (2019). Novel vaccine targeting colonic adenoma: a pre-clinical model. J Gastrointest Surg.

[149] Cross RS, Malaterre J, Davenport AJ (2015). Therapeutic DNA vaccination against colorectal cancer by targeting the MYB oncoprotein. Clin Transl Immunology.

[150] Pham T, Pereira L, Roth S (2019). First-in-human phase I clinical trial of a combined immune modulatory approach using TetMYB vaccine and Anti-PD-1 antibody in patients with advanced solid cancer including colorectal or adenoid cystic carcinoma: The MYPHISMO study protocol (NCT03287427). Contemp Clin Trials Commun.

[151] Mahmood U, Bang A, Chen YH (2021). A randomized phase 2 study of pembrolizumab with or without radiation in patients with recurrent or metastatic adenoid cystic carcinoma. Int J Radiat Oncol Biol Phys.

[152] Cleymaet R, Vermassen T, Coopman R, Vermeersch H, De Keukeleire S, Rottey S (2022). The therapeutic landscape of salivary gland malignancies-where are we now?. Int J Mol Sci.

[153] Dillon PM, Petroni GR, Horton BJ (2017). A phase II study of dovitinib in patients with recurrent or metastatic adenoid cystic carcinoma. Clin Cancer Res.

[154] Tchekmedyian V, Sherman EJ, Dunn L (2019). Phase II study of lenvatinib in patients with progressive, recurrent or metastatic adenoid cystic carcinoma. J Clin Oncol.

[155] Ho AL, Dunn L, Sherman EJ (2016). A phase II study of axitinib (AG-013736) in patients with incurable adenoid cystic carcinoma. Ann Oncol.

[156] Locati LD, Perrone F, Cortelazzi B (2016). A phase II study of sorafenib in recurrent and/or metastatic salivary gland carcinomas: Translational analyses and clinical impact. Eur J Cancer.

[157] Couvreur K, Celine J, Marlies B (2020). Efficacy and toxicity of sorafenib in patients with adenoid cystic carcinoma of the head and neck: a case series of five patients. Acta Clin Belg.

[158] Zheng S, Li H, Lin Y (2022). Treatment response to eribulin and anlotinib in lung metastases from rare perianal adenoid cystic carcinoma: a case report. Anticancer Drugs.

[159] Su N, Fang Y, Wang J (2022). Efficacy and safety of anlotinib in metastatic adenoid cystic carcinoma: a retrospective study. Transl Cancer Res.

[160] Hanna GJ, Ahn MJ, Muzaffar J (2023). A phase II trial of rivoceranib, an oral vascular endothelial growth factor receptor 2 inhibitor, for recurrent or metastatic adenoid cystic carcinoma. Clin Cancer Res.

[161] Ono J, Okada Y (2018). Study of MYB-NFIB chimeric gene expression, tumor angiogenesis, and proliferation in adenoid cystic carcinoma of salivary gland. Odontology.

[162] Chen C, Choudhury S, Wangsa D (2017). A multiplex preclinical model for adenoid cystic carcinoma of the salivary gland identifies regorafenib as a potential therapeutic drug. Sci Rep.

[163] Dai YH, Hung LY, Chen RY, Lai CH, Chang KC (2016). ON 01910.Na inhibits growth of diffuse large B-cell lymphoma by cytoplasmic sequestration of sumoylated C-MYB/TRAF6 complex. Transl Res.

[164] Sun B, Wang Y, Sun J (2021). Establishment of patient-derived xenograft models of adenoid cystic carcinoma to assess pre-clinical efficacy of combination therapy of a PI3K inhibitor and retinoic acid. Am J Cancer Res.

[165] Ladu S, Calvisi DF, Conner EA, Farina M, Factor VM, Thorgeirsson SS (2008). E2F1 inhibits c-Myc-driven apoptosis via PIK3CA/Akt/mTOR and COX-2 in a mouse model of human liver cancer. Gastroenterology.

[166] Hugo HJ, Saunders C, Ramsay RG, Thompson EW (2015). New insights on COX-2 in chronic inflammation driving breast cancer growth and metastasis. J Mammary Gland Biol Neoplasia.

[167] Kim DW, Oh DY, Shin SH (2014). A multicenter phase II study of everolimus in patients with progressive unresectable adenoid cystic carcinoma. BMC Cancer.

[168] Harvey RD, Carthon BC, Lewis C (2020). Phase 1 safety and pharmacodynamic study of lenalidomide combined with everolimus in patients with advanced solid malignancies with efficacy signal in adenoid cystic carcinoma. Br J Cancer.

[169] Zhang X, Lv QL, Huang YT, Zhang LH, Zhou HH (2017). Akt/FoxM1 signaling pathway-mediated upregulation of MYBL2 promotes progression of human glioma. J Exp Clin Cancer Res.

[170] Biyanee A, Yusenko MV, Klempnauer KH (2022). Src-family protein kinase inhibitors suppress MYB activity in a p300-dependent manner. Cells.

[171] Murray HC, Miller K, Brzozowski JS (2023). Synergistic targeting of DNA-PK and KIT signaling pathways in KIT mutant acute myeloid leukemia. Mol Cell Proteomics.

[172] Wong SJ, Karrison T, Hayes DN (2016). Phase II trial of dasatinib for recurrent or metastatic c-KIT expressing adenoid cystic carcinoma and for nonadenoid cystic malignant salivary tumors. Ann Oncol.

[173] Matsumura-Kimoto Y, Tsukamoto T, Shimura Y (2020). Serine-227 in the N-terminal kinase domain of RSK2 is a potential therapeutic target for mantle cell lymphoma. Cancer Med.

[174] Soncini D, Orecchioni S, Ruberti S (2020). The new small tyrosine kinase inhibitor ARQ531 targets acute myeloid leukemia cells by disrupting multiple tumor-addicted programs. Haematologica.

[175] Shirazi PT, Eadie LN, Heatley SL (2022). Exploring the oncogenic and therapeutic target potential of the MYB-TYK2 fusion gene in B-cell acute lymphoblastic leukemia. Cancer Gene Ther.

[176] Zhang Y, Li J, Zhong H (2021). The JAK2 inhibitor TG101209 exhibits anti-tumor and chemotherapeutic sensitizing effects on Burkitt lymphoma cells by inhibiting the JAK2/STAT3/c-MYB signaling axis. Cell Death Discov.

[177] Andersson MK, Mangiapane G, Nevado PT (2020). ATR is a MYB regulated gene and potential therapeutic target in adenoid cystic carcinoma. Oncogenesis.

[178] Zhou F, Zhang L, van Laar T, van Dam H, Ten Dijke P (2011). GSK3β inactivation induces apoptosis of leukemia cells by repressing the function of c-Myb. Mol Biol Cell.

[179] Albert V, Piendl G, Yousseff D (2022). Protein kinase C targeting of luminal (T-47D), luminal/HER2-positive (BT474), and triple negative (HCC1806) breast cancer cells in-vitro with AEB071 (Sotrastaurin) is efficient but mediated by subtype specific molecular effects. Arch Gynecol Obstet.

[180] Liu W, Wu M, Huang Z (2017). c-myb hyperactivity leads to myeloid and lymphoid malignancies in zebrafish. Leukemia.

[181] Ye Y, Yang X, Li F, Liu W, Zhang W, Huang Z

[182] De Dominici M, Porazzi P, Soliera AR (2018). Targeting CDK6 and BCL2 exploits the “MYB addiction” of Ph^+^ acute lymphoblastic leukemia. Cancer Res.

[183] Mitra P, Yang RM, Sutton J, Ramsay RG, Gonda TJ (2016). CDK9 inhibitors selectively target estrogen receptor-positive breast cancer cells through combined inhibition of MYB and MCL-1 expression. Oncotarget.

[184] Chen HX, Sharon E (2013). IGF-1R as an anti-cancer target--trials and tribulations. Chin J Cancer.

[185] Andersson MK, Åman P, Stenman G (2019). IGF2/IGF1R signaling as a therapeutic target in MYB-positive adenoid cystic carcinomas and other fusion gene-driven tumors. Cells.

[186] Andersson MK, Afshari MK, Andrén Y, Wick MJ, Stenman G (2017). Targeting the oncogenic transcriptional regulator MYB in adenoid cystic carcinoma by inhibition of IGF1R/AKT signaling. J Natl Cancer Inst.

[187] Pavlicek A, Lira ME, Lee NV (2013). Molecular predictors of sensitivity to the insulin-like growth factor 1 receptor inhibitor Figitumumab (CP-751,871). Mol Cancer Ther.

[188] Calvo E, Soria JC, Ma WW (2017). A phase I clinical trial and independent patient-derived xenograft study of combined targeted treatment with dacomitinib and figitumumab in advanced solid tumors. Clin Cancer Res.

[189] Parikh AS, Wizel A, Davis D (2022). Single-cell RNA sequencing identifies a paracrine interaction that may drive oncogenic notch signaling in human adenoid cystic carcinoma. Cell Rep.

[190] Eliasz S, Liang S, Chen Y (2010). Notch-1 stimulates survival of lung adenocarcinoma cells during hypoxia by activating the IGF-1R pathway. Oncogene.

[191] Brana I, Berger R, Golan T (2014). A parallel-arm phase I trial of the humanised anti-IGF-1R antibody dalotuzumab in combination with the AKT inhibitor MK-2206, the mTOR inhibitor ridaforolimus, or the NOTCH inhibitor MK-0752, in patients with advanced solid tumours. Br J Cancer.

[192] Thompson MA, Rosenthal MA, Ellis SL (1998). c-Myb down-regulation is associated with human colon cell differentiation, apoptosis, and decreased Bcl-2 expression. Cancer Res.

[193] Frech M, Teichler S, Feld C (2018). MYB induces the expression of the oncogenic corepressor SKI in acute myeloid leukemia. Oncotarget.

[194] Vrana JA, Decker RH, Johnson CR (1999). Induction of apoptosis in U937 human leukemia cells by suberoylanilide hydroxamic acid (SAHA) proceeds through pathways that are regulated by Bcl-2/Bcl-XL, c-Jun, and p21CIP1, but independent of p53. Oncogene.

[195] Claerhout S, Lim JY, Choi W (2011). Gene expression signature analysis identifies vorinostat as a candidate therapy for gastric cancer. PLoS One.

[196] Goncalves PH, Heilbrun LK, Barrett MT (2017). A phase 2 study of vorinostat in locally advanced, recurrent, or metastatic adenoid cystic carcinoma. Oncotarget.

[197] (2012). Calzada A, Todoerti K, Donadoni L, et al; AGIMM Investigators. The HDAC inhibitor Givinostat modulates the hematopoietic transcription factors NFE2 and C-MYB in JAK2(V617F) myeloproliferative neoplasm cells. Exp Hematol.

[198] Courdy C, Platteeuw L, Ducau C (2023). Targeting PP2A-dependent autophagy enhances sensitivity to ruxolitinib in JAK2^V617F^ myeloproliferative neoplasms. Blood Cancer J.

[199] Yusenko MV, Klempnauer KH (2022). Characterization of the MYB-inhibitory potential of the Pan-HDAC inhibitor LAQ824. BBA Adv.

[200] Kaundal B, Srivastava AK, Dev A, Mohanbhai SJ, Karmakar S, Roy Choudhury S (2020). Nanoformulation of EPZ011989 attenuates EZH2-c-Myb epigenetic interaction by proteasomal degradation in acute myeloid leukemia. Mol Pharm.

[201] Roe JS, Mercan F, Rivera K, Pappin DJ, Vakoc CR (2015). BET bromodomain inhibition suppresses the function of hematopoietic transcription factors in acute myeloid leukemia. Mol Cell.

[202] Bhagwat AS, Roe JS, Mok BYL, Hohmann AF, Shi J, Vakoc CR (2016). BET bromodomain inhibition releases the mediator complex from select cis-regulatory elements. Cell Rep.

[203] Lim SL, Damnernsawad A, Shyamsunder P (2019). Proteolysis targeting chimeric molecules as therapy for multiple myeloma: efficacy, biomarker and drug combinations. Haematologica.

[204] Rose AJ, Fleming MM, Francis JC (2024). Cell-type-specific tumour sensitivity identified with a bromodomain targeting PROTAC in adenoid cystic carcinoma. J Pathol.

[205] Liu Q, Liu H, Huang X (2023). A targetable MYBL2-ATAD2 axis governs cell proliferation in ovarian cancer. Cancer Gene Ther.

[206] Mandelbaum J, Shestopalov IA, Henderson RE (2018). Zebrafish blastomere screen identifies retinoic acid suppression of *MYB* in adenoid cystic carcinoma. J Exp Med.

[207] Zhou MJ, Yang JJ, Ma TY (2023). Increased retinoic acid signaling decreases lung metastasis in salivary adenoid cystic carcinoma by inhibiting the noncanonical Notch1 pathway. Exp Mol Med.

[208] Hanna GJ, ONeill A, Cutler JM (2021). A phase II trial of all-trans retinoic acid (ATRA) in advanced adenoid cystic carcinoma. Oral Oncol.

[209] Kasukabe T, Okabe-Kado J, Hozumi M, Honma Y (1994). Inhibition by interleukin 4 of leukemia inhibitory factor-, interleukin 6-, and dexamethasone-induced differentiation of mouse myeloid leukemia cells: role of c-myc and junB proto-oncogenes. Cancer Res.

[210] Yang H, Dai X, Ai Z (2019). MicroRNA-16 regulates myeloblastosis oncogene expression to affect differentiation of acute leukemia cells. Clin Lab.

[211] Bandyopadhyay A, Palepu S, Dhamija P (2023). Safety and efficacy of Vitamin D_3_ supplementation with imatinib in chronic phase- chronic myeloid leukaemia: an exploratory randomized controlled trial. BMJ Open.

[212] Al-Ali L, Al-Ani RJ, Saleh MM (2024). Biological evaluation of combinations of tyrosine kinase inhibitors with Inecalcitol as novel treatments for human chronic myeloid leukemia. Saudi Pharm J.

[213] Wilkins HR, Doucet K, Duke V, Morra A, Johnson N (2010). Estrogen prevents sustained COLO-205 human colon cancer cell growth by inducing apoptosis, decreasing c-myb protein, and decreasing transcription of the anti-apoptotic protein bcl-2. Tumour Biol.

[214] Salisbury TB, Morris GZ, Tomblin JK, Chaudhry AR, Cook CR, Santanam N (2013). Aryl hydrocarbon receptor ligands inhibit igf-ii and adipokine stimulated breast cancer cell proliferation. ISRN Endocrinol.

[215] Collins CT, Hess JL (2016). Role of HOXA9 in leukemia: dysregulation, cofactors and essential genes. Oncogene.

[216] Sonoda Y, Itoh M, Tohda S (2021). Effects of HOXA9 inhibitor DB818 on the growth of acute myeloid leukaemia cells. Anticancer Res.

[217] Gehringer F, Weissinger SE, Swier LJ, Möller P, Wirth T, Ushmorov A (2019). FOXO1 confers maintenance of the dark zone proliferation and survival program and can be pharmacologically targeted in burkitt lymphoma. Cancers.

[218] Zheng L, Feng Z, Tao S (2022). Destabilization of macrophage migration inhibitory factor by 4-IPP reduces NF-κB/P-TEFb complex-mediated c-Myb transcription to suppress osteosarcoma tumourigenesis. Clin Transl Med.

[219] McSheehy PM, Gervasoni M, Lampasona V, Erba E, D’Incalci M (1991). Studies of the differentiation properties of camptothecin in the human leukaemic cells K562. Eur J Cancer.

[220] Li F, Zhou J, Xu M, Yuan G (2018). Exploration of G-quadruplex function in c-Myb gene and its transcriptional regulation by topotecan. Int J Biol Macromol.

[221] Liu Q, Wang Q, Lv C (2021). Brucine inhibits proliferation of glioblastoma cells by targeting the G-quadruplexes in the c-Myb promoter. J Cancer.

[222] Zauli G, Voltan R, di Iasio MG (2011). miR-34a induces the downregulation of both *E2F1* and *B-Myb* oncogenes in leukemic cells. Clin Cancer Res.

[223] Sottile F, Gnemmi I, Cantilena S, D’Acunto WC, Sala A (2012). A chemical screen identifies the chemotherapeutic drug topotecan as a specific inhibitor of the B-MYB/MYCN axis in neuroblastoma. Oncotarget.

[224] Li Y, Zhao D, Zhang W (2023). A novel camptothecin derivative, ZBH-01, exhibits superior antitumor efficacy than irinotecan by regulating the cell cycle. J Transl Med.

[225] Okuno K, Garg R, Yuan YC, Tokunaga M, Kinugasa Y, Goel A (2022). Corrigendum: Berberine and oligomeric proanthocyanidins exhibit synergistic efficacy through regulation of PI3K-Akt signaling pathway in colorectal cancer. Front Oncol.

[226] Schmidt TJ, Klempnauer KH (2022). Natural products with antitumor potential targeting the MYB-C/EBPβ-p300 transcription module. Molecules.

[227] Klempnauer KH (2024). Transcription factor MYB as therapeutic target: current developments. Int J Mol Sci.

[228] Uttarkar S, Dassé E, Coulibaly A (2016). Targeting acute myeloid leukemia with a small molecule inhibitor of the Myb/p300 interaction. Blood.

[229] Clesham K, Walf-Vorderwülbecke V, Gasparoli L (2022). Identification of a c-MYB-directed therapeutic for acute myeloid leukemia. Leukemia.

[230] (2023). Tejera Nevado P, Tešan Tomić T, Atefyekta A, Fehr A, Stenman G, Andersson MK. Synthetic oleanane triterpenoids suppress MYB oncogene activity and sensitize T-cell acute lymphoblastic leukemia cells to chemotherapy. Front Oncol.

[231] Yusenko MV, Trentmann A, Andersson MK (2020). Monensin, a novel potent MYB inhibitor, suppresses proliferation of acute myeloid leukemia and adenoid cystic carcinoma cells. Cancer Lett.

[232] Lee YJ, Kang YR, Lee SY, Jin Y, Han DC, Kwon BM (2017). Ginkgetin induces G2-phase arrest in HCT116 colon cancer cells through the modulation of b-Myb and miRNA34a expression. Int J Oncol.

[233] Liu XM, Wang LG, Li HY, Ji XJ (1996). Induction of differentiation and down-regulation of c-myb gene expression in ML-1 human myeloblastic leukemia cells by the clinically effective anti-leukemia agent meisoindigo. Biochem Pharmacol.

[234] Chen F, Li L, Ma D (2010). Imatinib achieved complete cytogenetic response in a CML patient received 32-year indirubin and its derivative treatment. Leuk Res.

[235] Han SS, Han S, Kamberos NL (2014). Piperlongumine inhibits the proliferation and survival of B-cell acute lymphoblastic leukemia cell lines irrespective of glucocorticoid resistance. Biochem Biophys Res Commun.

[236] Yusenko M, Jakobs A, Klempnauer KH (2018). A novel cell-based screening assay for small-molecule MYB inhibitors identifies podophyllotoxins teniposide and etoposide as inhibitors of MYB activity. Sci Rep.

[237] Yusenko MV, Biyanee A, Frank D (2021). Bcr-TMP, a novel nanomolar-active compound that exhibits both MYB- and microtubule-inhibitory activity. Cancers.

[238] Köhler LHF, Reich S, Yusenko M (2022). A new naphthopyran derivative combines *c*-Myb inhibition, microtubule-targeting effects, and antiangiogenic properties. ACS Med Chem Lett.

[239] Köhler LHF, Reich S, Yusenko M (2023). Multimodal 4-arylchromene derivatives with microtubule-destabilizing, anti-angiogenic, and MYB-inhibitory activities. Cancer Drug Resist.

[240] Uttarkar S, Dukare S, Bopp B, Goblirsch M, Jose J, Klempnauer KH (2015). Naphthol AS-E phosphate inhibits the activity of the transcription factor Myb by blocking the interaction with the KIX domain of the coactivator p300. Mol Cancer Ther.

[241] Ramaswamy K, Forbes L, Minuesa G (2018). Peptidomimetic blockade of MYB in acute myeloid leukemia. Nat Commun.

[242] Joy ST, Henley MJ, De Salle SN (2021). A dual-site inhibitor of CBP/p300 KIX is a selective and effective modulator of Myb. J Am Chem Soc.

[243] Yusenko MV, Biyanee A, Andersson MK (2021). Proteasome inhibitors suppress MYB oncogenic activity in a p300-dependent manner. Cancer Lett.

[244] Walf-Vorderwülbecke V, Pearce K, Brooks T (2018). Targeting acute myeloid leukemia by drug-induced c-MYB degradation. Leukemia.

[245] Smith C, Touzart A, Simonin M (2023). Harnessing the MYB-dependent TAL1 5’super-enhancer for targeted therapy in T-ALL. Mol Cancer.

[246] Hu T, Chong Y, Cai B, Liu Y, Lu S, Cowell JK (2019). DNA methyltransferase 1-mediated CpG methylation of the miR-150-5p promoter contributes to fibroblast growth factor receptor 1-driven leukemogenesis. J Biol Chem.

[247] Daniel JP, Mesquita FP, Da Silva EL (2022). Anticancer potential of mebendazole against chronic myeloid leukemia: in silico and in vitro studies revealed new insights about the mechanism of action. Front Pharmacol.

[248] Sakka L, Delétage N, Chalus M (2017). Assessment of citalopram and escitalopram on neuroblastoma cell lines: cell toxicity and gene modulation. Oncotarget.

[249] Zhuo C, Xun Z, Hou W (2019). Surprising anticancer activities of psychiatric medications: old drugs offer new hope for patients with brain cancer. Front Pharmacol.

[250] Sumitani K, Kamijo R, Nagumo M (1997). Cytotoxic effect of sodium nitroprusside on cancer cells: involvement of apoptosis and suppression of c-myc and c-myb proto-oncogene expression. Anticancer Res.

[251] Magrinat G, Mason SN, Shami PJ, Weinberg JB (1992). Nitric oxide modulation of human leukemia cell differentiation and gene expression. Blood.

